# Electrostatic Co‐Assembly of Cyanine Pair for Augmented Photoacoustic Imaging and Photothermal Therapy

**DOI:** 10.1002/advs.202416905

**Published:** 2025-02-14

**Authors:** Haiqiao Huang, Yingnan Wu, Xin He, Yahang Liu, Jing‐Hui Zhu, Mingrui Gu, Danhong Zhou, Saran Long, Yahui Chen, Lei Wang, Mingle Li, Xiaoqiang Chen, Xiaojun Peng

**Affiliations:** ^1^ College of Materials Science and Engineering Shenzhen University Shenzhen 518060 P. R. China; ^2^ College of Physics and Optoelectronic Engineering Shenzhen University Shenzhen 518060 P. R. China; ^3^ Marshall Laboratory of Biomedical Engineering Shenzhen University Shenzhen 518060 P. R. China; ^4^ State Key Laboratory of Fine Chemicals Dalian University of Technology Dalian 116024 P. R. China

**Keywords:** co‐assembly, cyanine pair, j aggregates, photothermal therapy, photoacoustic imaging

## Abstract

Molecular phototheranostic dyes are of eminent interest for oncological diagnosis and imaging‐guided phototherapy. However, it remains challenging to develop photosensitizers (PSs) that simultaneously integrate high‐contrast photoacoustic imaging and efficient therapeutic capabilities. In this work, a supramolecular strategy is employed to construct a molecular pair phototheranostic agent via the direct self‐assembly of two cyanines, C5TNa (anionic) and Cy‐Et (cationic). The Coulombic interactions between C5TNa and Cy‐Et facilitate the formation of a complementary cyanine pair (C5T‐ET) and the creation of supramolecular CT‐J‐type aggregates in water. This complementary cyanine pair (C5T‐ET) results in completely quenched fluorescence and significantly enhances nonradiative deactivation (≈22 ps), leading to a 3.3‐fold increase in photothermal conversion efficiency and a 7.1‐fold enhancement in photoacoustic response compared to indocyanine green (ICG). As a result, the J‐type aggregate cyanine pair (C5T‐ET) demonstrates high photoacoustic imaging capability and remarkable antitumor phototheranostic efficacy in vivo, highlighting its potential for clinical applications. This work provides strong experimental evidence for the superior performance of the complementary cyanine pair (C5T‐ET) in enhancing photosensitization and photoacoustic response. It is believed that this strategy will propel the advancement of controllable dye J‐aggregates and contribute to the practical implementation of photoacoustic imaging and phototherapy in vivo.

## Graphical abstract

A molecular pair phototheranostic agent is developed through the supramolecular assembly of two cyanine dyes, C5TNa (anionic) and Cy‐Et (cationic). The complementary cyanine pair, C5T‐ET, self‐assembles into J‐type aggregates in aqueous environments. The C5T‐ET complex demonstrates a 3.3‐fold increase in photothermal efficiency and a 7.1‐fold enhancement in photoacoustic response, offering significant improvements over the conventional agent ICG.

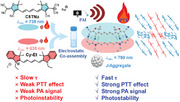

## 1 Introduction

Photothermal therapy (PTT) has made significant progress in cancer therapeutics, thanks to its exceptional non‐invasive nature and spatiotemporal resolution.^[^ ^1^ ^]^ It is widely recognized as a promising therapeutic approach with great potential. The development of high‐performance photothermal agents (PTAs) and drugs has become a critical area of research. Near‐infrared (NIR) organic molecular dyes, owing to their unique photophysical properties, superior tissue penetration, and excellent biocompatibility and biodegradability, have garnered considerable attention in biomedical imaging and phototherapy.^[^ ^2^ ^]^ Under NIR laser irradiation, these dyes can efficiently produce cytotoxic reactive oxygen species (ROS) and/or heat, which are used in photodynamic therapy (PDT) and PTT, respectively, to mightily eradicate cancer cells.^[^ ^3^, ^4^ ^]^ Over the past few decades, certain representative molecular dyes, such as boron‐dipyrromethene (BODIPY) and cyanine dyes (Cys), have been extensively studied for their anticancer potential.^[^ ^5^ ^]^ These dyes feature molecular cores with extensive π‐conjugation, allowing for absorption and emission at longer wavelengths. In efforts to enhance the performance of these organic PTAs, researchers have often focused on increasing molecular flexibility to improve photothermal effects. However, this approach has a notable drawback: it may reduce fluorescence efficiency, thus impairing the ability for fluorescence‐guided imaging.

Photoacoustic imaging (PAI) has opened new avenues for the advancement of theranostic imaging technologies.^[^ ^6^, ^7^ ^]^ In PAI, laser pulses excite light‐absorbing agents, with the absorbed laser energy being converted into heat by endogenous or exogenous contrast agents within the tissue. This results in transient thermoelastic expansion, generating corresponding acoustic waves, which are subsequently detected and analyzed by ultrasound transducers to form high‐resolution images. PAI is a novel biomedical imaging method that facilitates in vivo imaging with significant tissue penetration while maintaining high image contrast and spatial resolution.^[^ ^8^ ^]^ In comparison to fluorescence imaging, PAI offers superior tissue penetration (up to 5–6 cm), making it particularly advantageous for imaging deeper tissues, with notable clinical applications. Consequently, the development of organic NIR PTAs with efficient PAI capabilities, enhanced photothermal effects, and excellent biocompatibility is of paramount importance.^[^ ^9^ ^]^

Traditional strategies for extending the absorption wavelength of dyes typically involve elongating conjugated chains or incorporating large conjugated moieties. However, these approaches often introduce challenges such as stability issues and poor water solubility.^[^ ^10^, ^11^, ^12^, ^13^, ^14^ ^]^ In contrast to individual molecules, when dyes undergo J‐aggregation, a significant redshift in the absorption wavelength occurs, presenting a promising strategy for designing NIR fluorescent photosensitizers (PSs).^[^ ^15^ ^]^ This strategy circumvents the limitations associated with the complex design and labor‐intensive synthesis of long‐wavelength monomers. In recent years, there has been remarkable progress in the study of NIR aggregates, expanding their applications in biomedical imaging and therapy.^[^ ^16^ ^]^ These pioneering studies have offered significant insights into the design of NIR J‐aggregated dyes. However, most research has focused on molecules based on traditional scaffolds, such as BODIPY or Cys, which exhibit inherent design limitations. For example, the aggregation of these compounds was highly dependent on concentration, often requiring the presence of external salts or encapsulation in a specific medium. Furthermore, aggregation of these molecules typically relies on strategies like covalent dimerization, functional group conjugation, solvent‐induced interactions, or encapsulation within carriers—each presenting its own set of challenges and limitations (Table S1, Supporting Information).^[^ ^17^ ^]^ Currently, available molecular libraries remain relatively limited, and structures with large scaffolds often require complex design, tedious synthesis, and purification processes. Thus, the development of more streamlined strategies for designing NIR photothermal materials remains a critical, unsolved challenge.

**Figure 1:**
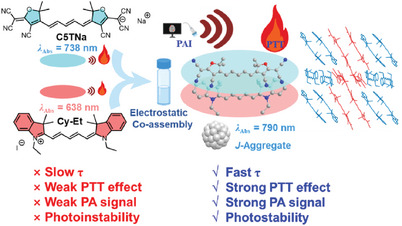
Schematic illustration of the design concept for molecular pairs of C5T‐ET based electrostatic interactions and its enhanced highly efficient PAI and PTT. Figure created in https://BioRender.com.

In this study, an anionic pentamethyl‐derivative of cyanine dye (C5TNa) was selected as the model dye, and molecular pairs of C5T‐X with various cationic pentamethyl‐cyanine and heptamethyl‐cyanine (Cy7) dyes were assembled through electrostatic interactions. The results revealed that the counterion exerted a pivotal influence on both the molecular assembly and the optical properties of these compounds. Compared to pure anionic C5TNa and cationic Cy‐Et dyes, the molecular pair C5T‐ET exhibited enhanced photoacoustic (PA) signals and photothermal effects (**Figure** **1**). More importantly, C5T‐ET enabled high‐contrast in vivo tumor visualization via PAI. Finally, the study demonstrated the potential of C5T‐ET for cancer PTT.

## 2 Results and Discussion

The molecular structures and synthetic routes are provided in Scheme S1 (Supporting Information). The products were characterized using nuclear magnetic resonance (NMR) and high‐resolution mass spectrometry (HRMS) (Figures S22–S24, Supporting Information). Single‐crystal X‐ray diffraction was utilized to determine the structures of C5TNa and C5T‐ET.

In this study, 2‐dicyanomethylene‐3‐cyano‐4,5,5‐trimethyl‐2,5‐dihydrofuran (TCF) and N‐ethyl‐2,3,3‐trimethylindolenine (EID) were utilized as terminal groups for anionic and cationic cyanines, respectively. The TCF terminal group, known for its extensive electronic delocalization, was initially developed as an efficient electron acceptor for dipolar dyes. In contrast, the EID terminal group, characterized by pronounced electronic localization, was originally developed as an effective electron donor for dipolar dyes.

**Figure 2:**
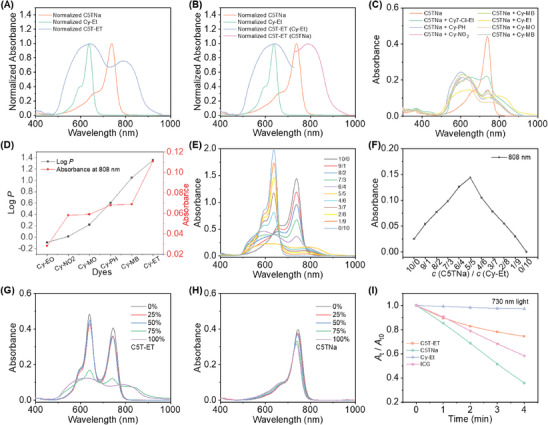
A) Normalized absorption spectra of C5TNa, Cy‐Et, and C5T‐ET in water. B) Normalized absorption spectra of C5TNa, Cy‐Et, C5T‐ET (part of Cy‐Et), and C5T‐ET (part of C5TNa) in water. (c = 2.0 µm) C) The absorption spectra of Cy5s or Cy7 (c = 2.0 µm) with C5TNa (c = 2.0 µm) in Water. D) The log P and the absorbance at 808 nm Cy5s (c = 2.0 µm) with C5TNa (c = 2.0 µm) in Water. E) absorbance of C5TNa mixed with different molar ratios of Cy‐Et in water. F) Job's plot for evaluating the chelating ratio of C5TNa to Cy‐Et. The total concentration of C5TNa and Cy‐Et was 10.0 µm. Absorption spectra of C5T‐ET G) and C5TNa H) in MeOH‐H_2_O solutions with varied *f*_water_ (c = 2.0 µm). I) Relationship between the absorbance at the maximum absorption wavelength/the absorbance A0 of C5T‐ET, C5TNa, Cy‐Et, and ICG (10.0 µm) in water at different irradiation times (730 nm, 0.1 W cm^−2^).

To investigate the optical properties of C5TNa and Cy‐Et, their UV–Vis absorption and fluorescence spectra were recorded in ultrapure water. Figures S1 and S2 (Supporting Information) present the UV–Vis and fluorescence spectra of C5TNa and Cy‐Et in ethanol (EtOH) and methanol (MeOH). As shown in Figure S1 (Supporting Information), Cy‐Et exhibits a sharp and intense absorption at 638 nm (*ε* = 22.6 × 10^4^ m ^−1^ cm^−1^) and strong fluorescence at 657 nm (*Φ*_F_ = 9.7%) in water (Figure S1D, Supporting Information). Meanwhile, C5TNa shows sharp and intense absorption at 738 nm (*ε* = 18.7 × 10^4^ m ^−1^ cm^−1^) and strong fluorescence at 762 nm (*Φ*_F_ = 7.6%) in water (Figure S1E, Supporting Information). Interestingly, as shown in **Figure** 2A–C, when C5TNa and Cy‐Et are mixed to form C5T‐ET, the resulting spectra in water are notably different. Under the same conditions, C5TNa and Cy‐Et remain in a single‐molecule state in water, with their absorption peaks appearing as independent and sharp cyanine dye peaks. In contrast, C5T‐ET exhibits two significantly broadened absorption peaks: one at 638–644 nm for Cy‐Et and another at 790 nm for C5T, with the latter showing a substantial redshift of 52 nm compared to the C5TNa single‐molecule spectrum. These data strongly suggest the formation of J‐type aggregates in the C5T component of C5T‐ET in water, likely induced by the presence of a hydrophobic cationic cyanine counterion. Notably, the emission of C5T‐ET in MeOH and EtOH is similar to that of C5TNa, with emission spectra and intensities essentially consistent between C5T‐ET and C5TNa in these solvents. However, in water, the fluorescence intensity of C5T‐ET is undetectable (Figure S2D–F, Supporting Information), indicating quenched fluorescence. This quenching is likely attributed to the formation of molecular pair aggregates accompanied by strong intermolecular charge transfer (CT),^[^ ^5^, ^16^ ^]^ which could enhance PA emission.

Next, we tested five others cationic pentamethine cyanines with different substituents on the nitrogen atom (N) of the indoles. Surprisingly, no new peaks appeared with the addition of other cations. At the same time, we also investigated the mixed assembly of the cationic Cy7 dye and the anionic C5TNa cyanine dye in water. We observed a broad absorption peak only in the range of 500–680 nm, indicating that H‐aggregation occurs exclusively after the self‐assembly of the two dyes. Instead, the absorption peaks of Cy‐Et and C5TNa decreased, and a tail peak emerged above 800 nm (Figures 2C and S3, Supporting Information). Based on these observations, we hypothesize that the emergence of the new peak at 790 nm in C5T‐ET may be related to the fat solubility of the cationic Cys. We further measured the oil‐water partition coefficient (Log*P*) of all tested cationic Cys and plotted these values against the absorption of the mixed molecules at 808 nm. The Log*P* values of the cationic Cys are shown in Table S2 (Supporting Information). Our findings reveal that the higher Log*P* of a cationic cyanine dye, the greater the absorption of the mixed molecules pair at 808 nm (Figure 2D), indicating a linear correlation between the absorbance of cationic/anionic cyanine dye complexes in water and Log*P* of the cationic cyanine dye.

Then, the formation of a stable 1:1 complex between C5TNa and Cy‐Et, referred to as C5T‐ET, was confirmed using a Job's plot curve (Figures 2E,F and S4A,B, Supporting Information). To monitor the evolution of the J‐aggregates, we investigated the absorption and fluorescence spectra of C5TNa and C5T‐ET in MeOH‐H_2_O solutions with varying water fractions (*f*_water_). As illustrated in Figure 2G, when the water content ranged from 0–50%, the absorption bands at 640 and 746 nm of C5T‐ET exhibited only a slight decrease. However, at 75–100% water, the band at 640 nm underwent a sharp decrease, accompanied by broadening, while the band at 746 nm red‐shifted by 52 nm to 790 nm. This behavior indicates that C5T‐ET self‐assembles via *π*–*π* stacking, which affects the π‐π electronic transitions of the C5T core. In contrast, the absorption of C5TNa in MeOH‐H_2_O solutions showed only a slight decrease, accompanied by a blue shift (Figure 2H). The observed bathochromic shifts in the absorption bands of C5T‐ET suggest J‐type aggregation in MeOH‐H_2_O solvent mixtures. The fluorescence emission spectrum of C5T‐ET, when excited at 740 nm in pure MeOH, showed an emission peak at 768 nm (Figures S1F and S4C, Supporting Information). The intensity of the emission band of C5T‐ET diminished as water was added to the MeOH solution, with complete quenching of fluorescence observed at 100% water. This observation suggests that the addition of water to a MeOH solution of C5T‐ET induces self‐assembly via strong π–π stacking interactions of the C5T core, leading to quenching of the electronic relaxation transitions of the π‐conjugated core. Similarly, fluorescence studies of C5TNa in MeOH with varying water content were performed (Figure S4D, Supporting Information). Upon excitation at 740 nm, C5TNa exhibited an emission peak at 768 nm. The gradual addition of water to MeOH led to a steady reduction in emission intensity, along with a red shift of the peak to 760 nm in pure water. In addition, the observation of the Tyndall effect for C5T‐ET in water confirmed the successful formation of colloidal nanoparticles, which was further supported by Transmission Electron Microscopy (TEM) and Dynamic Light Scattering (DLS) measurements (Figure S5, Supporting Information). The hydrodynamic diameter of C5T‐ET assemblies in water was found to be ≈159 nm, indicating the successful construction of C5T‐ET nanoassemblies. TEM images revealed that the C5T‐ET aggregates exhibited a spherical morphology with a uniform size of ≈101 nm.

Moreover, to thoroughly investigate the impact of counterions on the aggregation behaviors and optical properties, we performed absorption spectroscopy at various concentrations. As shown in Figure S6 (Supporting Information), the absorbance of C5T‐ET at its maximum absorption wavelength displayed excellent linearity with concentration in the range of 2.0–30.0 µm. In contrast, C5TNa and Cy‐Et did not exhibit a linear relationship between absorbance and concentration, as deviations from linearity were observed when their concentrations exceeded 20.0 and 15.0 µm, respectively. These results suggest that the J‐aggregation of C5T‐ET in water is not significantly affected by dye concentration within the 2.0–30.0 µm range. In contrast, H‐aggregation occurs when the concentrations of C5TNa and Cy‐Et exceed 20.0 µm in water.

As mentioned earlier, the photobleaching properties of polymethine Cys limit their broader applications in the biomedical field. In this study, we systematically investigated the photostability of our designed dyes. As shown in Figures 2I and S7 (Supporting Information), C5T‐ET exhibited significantly greater stability compared to ICG and C5TNa when subjected to four minutes of 730 nm laser irradiation at a power density of 0.1 W cm^−2^. After 4 min of irradiation, C5T‐ET retained over 74% of its initial absorbance, while ICG and C5TNa retained less than 60%. A similar trend was examined under 808 nm laser irradiation under the same conditions (Figure S7, Supporting Information). Furthermore, the absorption spectra of different compound solutions before and after illumination indicated that the shape of the absorption peak of C5T‐ET remains largely unchanged, with only a moderate reduction in intensity. This suggests that molecular aggregation is not significantly affected by laser irradiation. These findings indicate that our designed complementary cyanine salt, C5T‐ET, possesses significantly improved photostability, making it more suitable for subsequent biological applications. Given the low fluorescence quantum yield of C5T‐ET, it is reasonable to speculate that the absorbed light energy is more efficiently converted into triplet excited states or heat.

**Figure 3:**
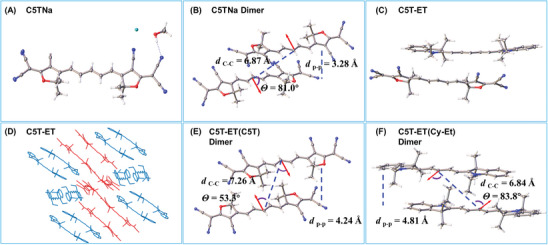
A) Crystal structures of C5TNa (CCDC no. 2407640). B) The packing motifs of aggregated dimers of C5TNa, with transition dipoles of S_1_ indicated by red arrows. The transition dipoles of S_1_ were computed by the time‐dependent density functional theory approach at the level of B3LYP/6‐311G(d). C) Co‐growing single crystals of C5T‐ET (C5T and Cy‐Et molar ratio 1:1) (CCDC no. 2407641). D) molecular packing of C5T‐ET, the part of Cy‐Et marked in cyan and part of C5T marked in red. Packing motifs of aggregated dimers of E) C5T‐ET (part of C5T) and F) C5T‐ET (part of Cy‐Et) with transition dipoles of S_1_ denoted as red arrows.

The aggregation behavior of C5TNa and C5T‐ET was investigated in detail due to their promising characteristics. Single crystals of both compounds were successfully obtained via vapor diffusion from a diethyl ether/MeOH solution at room temperature. As seen in **Figures** **3**A and S8A (Supporting Information), C5TNa displays a large conjugate plane, which is consistent with its long absorption and emission wavelengths (>740 nm). Additionally, the molecular packing of C5TNa shows a slip‐stack arrangement, with an intermolecular π–π distance (d_p‐p_) of 3.28 Å and a center‐to‐center distance (d_C‐C_) of 6.87 Å (Figures 3B and S8B, Supporting Information). Notably, the angle (θ) between the transition dipoles and the interconnected axis is 81.0° for C5TNa, which is much larger than the critical value of 54.7°, favoring the formation of H‐aggregates.

For the complementary cyanine pair, C5T‐ET, the refined structure is illustrated in Figure 3C (additional details can be found in Figure S8C, Supporting Information). The packing arrangement of C5T‐ET demonstrates a highly ordered molecular structure, with both Cy‐Et and C5T molecules arranged in a slip‐stack configuration (Figure 3D). Upon further analysis of the eutectic structure, it was observed that there is an angle between the molecular planes of Cy‐Et and C5T. Additionally, the nitrogen in the pyrrole ring of Cy‐Et and the furan ring of C5T are positioned back‐to‐back. This arrangement is likely driven by electrostatic interactions, where the nucleophilic part of Cy‐Et is closer to the electrophilic part of C5T, conferring increased chemical stability to the C5T‐ET molecular pair. Further investigation into the packing structures of the Cy‐Et and C5T dimers within C5T‐ET revealed distinct differences. As illustrated in Figures 3E, F and S8D, E (Supporting Information), the d_p‐p_ and d_C‐C_ are 4.81 and 6.84 Å for Cy‐Et, and 4.24 and 7.26 Å for C5T, respectively. The angles between the transition dipoles and the interconnected axis are 83.8° for Cy‐Et and 53.3° for C5T. These results suggest that Cy‐Et molecules form extended H‐aggregates, while C5T molecules form extended J‐aggregates, as anticipated. These findings not only highlight the differences in aggregation behavior between C5TNa and C5T‐ET, but also provide valuable insights into their structural dynamics, which are essential for understanding their photophysical properties and their potential in photothermal and PA applications.

**Figure 4:**
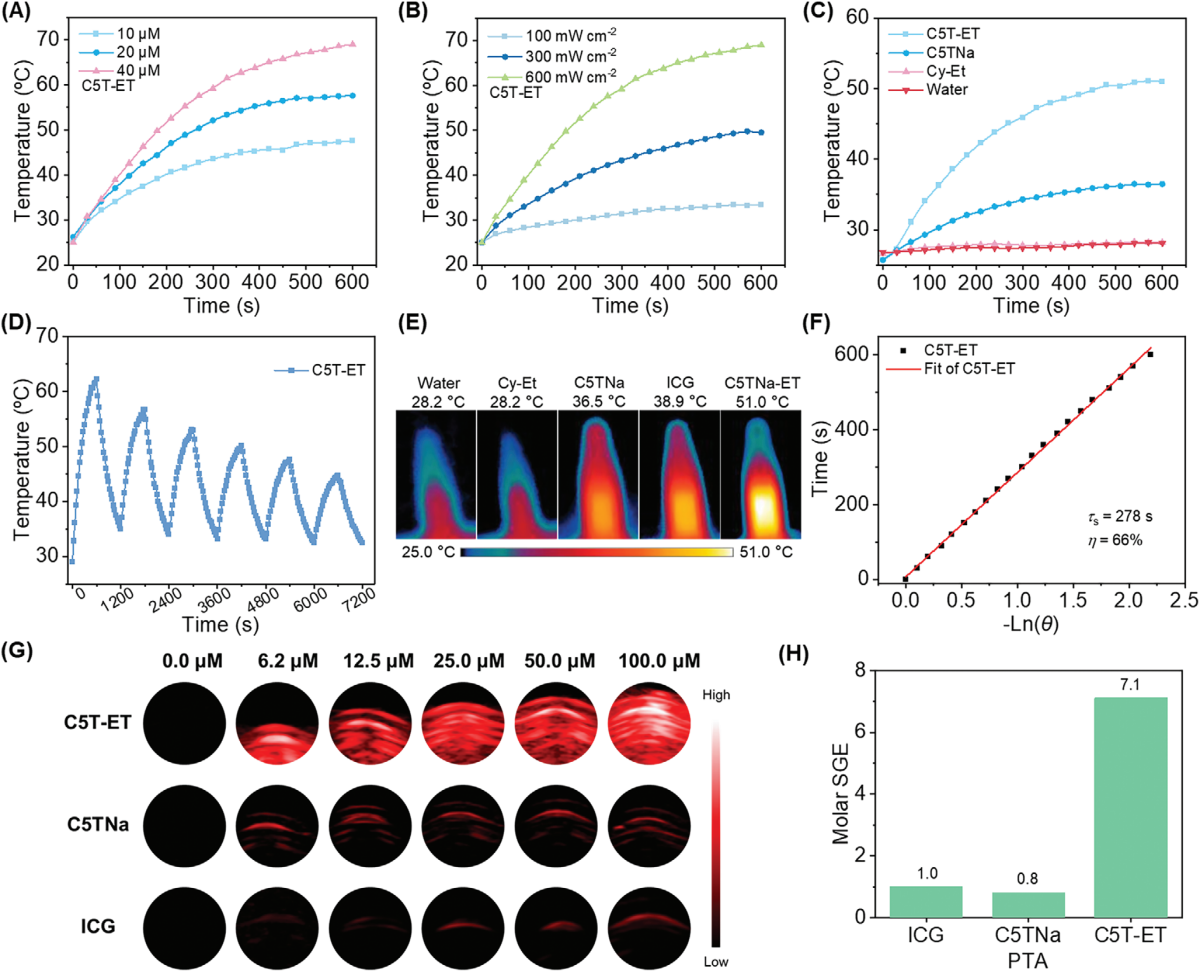
A) Photothermal conversion of C5T‐ET at different concentrations (10.0–40.0 µm) under 808 nm laser irradiation (0.6 W cm^−2^). B)Photothermal conversion of C5T‐ET (40.0 µm) under 808 nm laser irradiation with different exposure intensities (0.1–0.6 W cm^−2^). C) Photothermal conversion of C5T‐ET, C5TNa, Cy‐Et (20.0 µm) and water under 808 nm laser irradiation (0.6 W cm^−2^). D) Photothermal stability of C5T‐ET (20.0 µm) during five cycles of heating‐cooling processes. E) Photothermal images of water, Cy‐Et, C5TNa, ICG, and C5T‐ET (20.0 µm) under 808 nm laser irradiation (0.6 W cm^−2^, 10 min). E) Heat‐transferring time constants (τ_s_) determined by time−temperature data from the cooling period, and PCE calculation of C5T‐ET. G) In vitro PA images of C5T‐ET, C5TNa, and ICG at different concentrations (0–100.0 µm) in water. Images of C5T‐ET were collected at 780 nm photoexcitation; images of C5TNa were collected at 760 nm photoexcitation; images of ICG were collected at 772 nm photoexcitation. H) PA SGE at constant molarity, showing relative dye “loudness” (ICG set to 1).

In light of the above findings, the J‐aggregates of C5T‐ET may prove to be efficient NIR absorbing agents for PAI and PTT, due to their strong absorption beyond 790 nm and extremely low fluorescence quantum yield. We next investigated the photothermal and PA properties of ICG, C5TNa, and C5T‐ET. The photothermal effect of C5T‐ET in water was assessed by systematically varying its concentration and the energy density of the 808 nm laser. As illustrated in **Figure** **4**A,B, the resulting temperature of the solutions increased proportionally with both the concentration of C5T‐ET and the optical density. Upon irradiation with an 808 nm (0.6 W cm^−2^) laser for 10 min, the temperature of a 20.0 µm solution of C5T‐ET rose significantly from 25 to 51 °C (ΔT = 26 °C; Figure 4C). Notably, C5T‐ET demonstrated relatively excellent thermal and photostability, exhibiting minimal degradation after five cycles of heating and cooling under laser irradiation (Figure 4D). In addition, based on solution photographs (Figure S9A, Supporting Information), C5T‐ET exhibited only a slight decrease after 10 min of irradiation, whereas C5TNa and ICG showed significant reductions under the same conditions. These results suggest that C5T‐ET maintains relatively superior photostability during PTT processes. Photothermal imaging of C5T‐ET, C5TNa, ICG, and PBS confirmed the superior PTT effect of C5T‐ET (Figure 4E). Furthermore, the photothermal conversion efficiency (PCE, *η*) of C5T‐ET was calculated to be 66% (Figure 4F), significantly surpassing that of ICG (20.0%) and C5TNa (38%) (Figure S9B–D, Supporting Information).

Building on the extended NIR absorption and superior photothermal conversion properties of C5T‐ET, we next investigated whether the PA effect could be enhanced in vitro. As shown in Figure 4G, C5T‐ET exhibited the strongest PA signals across a concentration range from 0.0 to 100.0 µm. Furthermore, a robust linear relationship was observed between C5T‐ET concentration and PA intensity (Figure S10A, Supporting Information). The PA spectra of C5T‐ET were recorded by measuring the PA intensity at different wavelengths from 700 to 900 nm. Consistent with its outstanding photothermal effect, C5T‐ET generated significantly stronger PA amplitudes than either C5TNa or ICG. In particular, C5TNa produced a weaker PA signal (Figures 4G and S10, Supporting Information). These results demonstrated the feasibility of performing quantitative analysis based on PA signal intensity. As shown in Figure S10G (Supporting Information), the PA spectra closely mirrored the absorption profile, indicating that the PA signal originated from the absorption of the NIR chromophores. To robustly quantify the PA signal generation efficacy (SGE), the Thorn‐Threshold method was employed.^[^ ^18^ ^]^ We selected an intermediate concentration of 25.0 µm, which was linear for all species (ICG set to 1). Notably, the PA intensity of C5T‐ET was over 7.1 times higher than that of ICG (and higher than C5TNa), indicating its potential for clinical PAI studies (Figure 4H). Thus, the excellent PA response behavior suggests that C5T‐ET could potentially be applied for guiding in vivo PTT.

To elucidate the mechanism behind the formation of C5T‐ET dimmers and their unusual photophysical behavior, we theoretically investigated the electrostatic potential surface (ESP) of Cy‐Et and C5T and the excited states of both the monomer and complementary cyanine salts. These investigations were based on their single‐crystal structures in the aqueous phase, and density functional theory (DFT) and time‐dependent density functional theory (TD‐DFT) calculations were performed at the B3LYP/6‐311G(d) theoretical level using the Gaussian 16 program.

**Figure 5:**
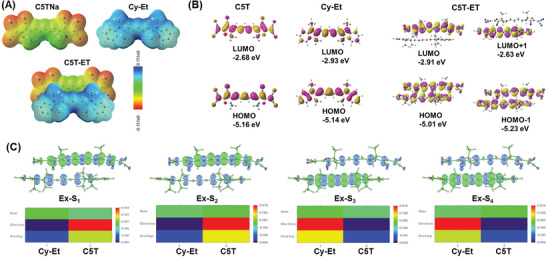
A) Mapped with the surface electrostatic potential of C5T and Cy‐Et (top) and the stimulated map of molecular pairs of C5T‐ET based electrostatic interactions (bottom). B) The frontier molecular orbital profiles of C5T, Cy‐Et, and C5T‐ET were calculated by using DFT (B3LYP/6‐31G^*^). C) Hole and electron distributions of S_1_ – S_4_ excited state in C5T‐ET. The blue and green represent the hole and electron distributions, respectively. Heat maps of the hole‐electron distributions of C5T‐ET at S_1_ – S_4_ state.

The ESP maps of the Cy‐Et and C5T dyes are shown in **Figure** **5**A, where the blue areas indicate regions of low electron density (with positive ESP), green represents normal electron density, and red areas suggest high electron density (with negative ESP). For the Cy‐Et molecule, the hydrogen atoms at its molecular surface led to the positive ESP and are electrophilic. For the C5T molecule, the nitrogen atoms in the terminal cyanide groups bear long pair electrons, displaying negative ESP, and are nucleophilic. Consequently, there is a strong electrostatic attraction between C5T and Cy‐ET, forming a C5T‐ET molecular pair, which was confirmed through single‐crystal analysis. The plain cyanine body of C5T‐ET promotes the formation of J‐aggregates between C5T‐ET dimers via π–π interaction, further enhancing its photophysical properties.

The calculated absorption spectra of Cy‐Et and C5T show the S_0_→S_1_ transition at 612 nm for Cy‐Et with an oscillator strength (*f*) of 2.3466, and 709 nm for C5T with a *f* of 2.6679 (Figure 5B). In contrast, the calculated absorption spectrum of C5T‐ET (Figures 5B and S11, and Table S3, Supporting Information) shows the S_0_→S_1_ transition at 762 nm with a lower *f* of 0.9875, while the strong transition is of S_0_→S_3_ at 646 nm with a higher *f* of 1.4839. Compared to C5T, the S_0_→S_1_ transition in C5T‐ET is red‐shifted by 53 nm, which closely matches the experimental absorption spectra in Figure 2A. The photophysical properties of the monomer and molecules pair are calculated by using TD‐DFT and listed in Table S3 (Supporting Information). Fluorescence occurs when a molecule emits photons during its transition from the lowest vibrational energy level of the first excited singlet state (S_1_) to the vibrational energy levels of the ground state (S_0_). As shown in Table S3 (Supporting Information), the calculated S_1_→S_0_ emissions of Cy‐Et and C5T are at 655 and 726 nm, respectively, with large *f* of 2.3372 and 2.6417. However, the S_1_→S_0_ emission for C5T‐ET is smaller (0.1855) than that of Cy‐Et and C5TNa, which aligns perfectly with the experimental results. This observation further suggests that the self‐assembled C5T‐ET molecule pair facilitates energy dissipation through non‐radiative transition pathways.

To further investigate the nature of the excited states in C5T‐ET, we visualized their hole and electron distributions using the Multiwfn program. As depicted in Figure 5C and Table S4 (Supporting Information), the S_1_ and S_4_ states of C5T‐ET maintain inter‐CT characteristics. For example, the electrons in the first excited state are concentrated on part of the C5T, while the holes are distributed across both C5T and Cy‐Et. This indicates a combination of local excitation (LE) in C5T and CT from Cy‐Et to C5T in the first excited state. These findings further confirm that CT transitions of C5T‐ET. The π–π interactions between C5T‐ET molecular pairs drive the formation of CT J‐aggregates.

Next, the fluorescence lifetimes of Cy‐Et, C5TNa, and C5T‐ET were determined using time‐correlated single‐photon counting at their respective excitation wavelengths in water. As illustrated in Figure S12 (Supporting Information), the fluorescence lifetimes of Cy‐Et and C5TNa in water were found to be 0.472 and 0.523 ns, respectively. However, the fluorescence lifetime of C5T‐ET could not be measured, suggesting that the strong interactions within the C5T‐ET pairs promote rapid non‐radiative decay from the excited singlet state. In addition, we also measured the fluorescence lifetime of C5T‐ET in MeOH (Figure S12, Supporting Information). Similar to the fluorescence spectra in solution, C5T‐ET exhibited the same fluorescence performance as C5TNa, indicating that C5T‐ET remains non‐aggregated in MeOH.

**Figure 6:**
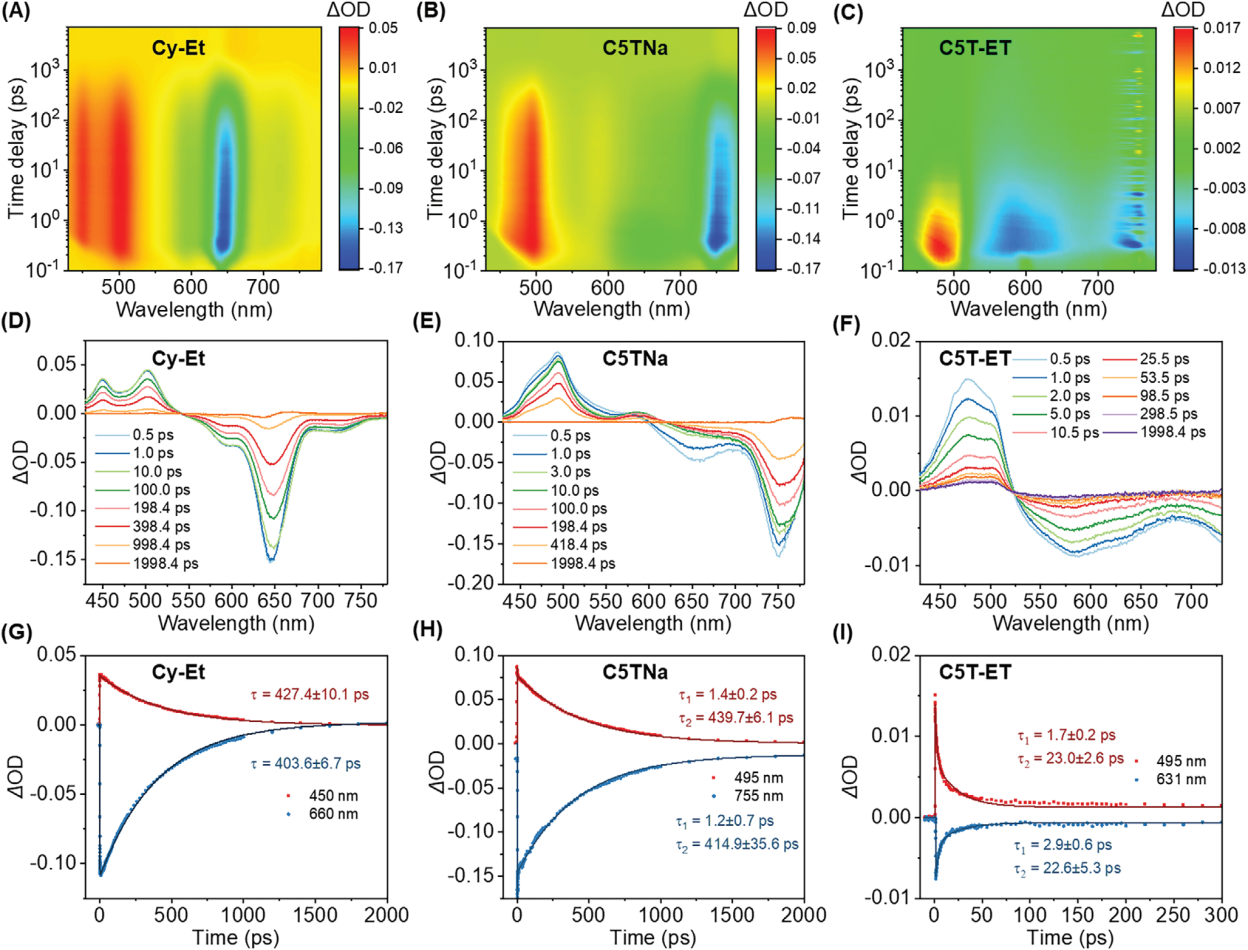
Mechanistic studies using fs‐TA spectrum. A–C) Fs‐TA mapping of Cy‐Et, C5TNa, and C5T‐ET in water. D–F) The fs‐TA spectra of Cy‐Et, C5TNa, and C5T‐ET at selected decay times. G–I) Extracted kinetic curves at representative wavelengths of Cy‐Et, C5TNa, and C5T‐ET.

To further explore the exciton dynamics of Cy‐Et, C5TNa, and C5T‐ET, femtosecond transient absorption (fs‐TA) pump‐probe spectroscopy was performed. As shown in **Figure** **6**, the cationic dye exhibits excited‐state absorption (ESA) within the 430–550 nm range, along with a negative signal attributed to ground‐state bleaching (GSB) and stimulated emission (SE) from 550–750 nm. Similarly, the anionic dye shows ESA in the 450–550 nm range and a combined GSB and SE signal spanning 600–780 nm. Both dyes exhibit extended decay lifetimes of over 400 ps. However, the anionic dye displays an additional rapid decay component with a lifetime of less than 2 ps, likely due to fast thermal deactivation from the singlet state. When combined, the cation‐anion interactions in C5T‐ET significantly accelerate the excited‐state decay, shortening the lifetime from hundreds of picoseconds (≈400 ps) to tens of picoseconds (≈22 ps), and all the fitted time constants are listed in Table S5 (Supporting Information). This is accompanied by an increased proportion of the rapid nonradiative deactivation component (with a lifetime > 2 ps) at the characteristic peak. These findings suggest that the molecular pairs in C5T‐ET effectively enhance nonradiative deactivation. According to literature reports, ultrafast excited‐state decay to the ground state enables multiple S_0_→S_1_→S_0_ cycles per pulse, contributing to outstanding photostability and excellent SGE, which are ideal for reliable quantification in NIR optoacoustic imaging.^[^ ^18^ ^]^ In this study, the J‐aggregate C5T‐ET, formed through electrostatic self‐assembly, exhibits an ultra‐fast S_1_→S_0_ relaxation, accelerating the transition compared to the single molecule C5TNa. This results in an 8.8‐fold stronger PA signal and enhanced photostability. The ultra‐fast decay of the excited state to the ground state contributes to the excellent photostability, outstanding photothermal properties, and high SGE of C5T‐ET in NIR PAI.

Driven by its high PCE, NIR absorption, and outstanding photothermal stability, C5T‐ET‐mediated PTT was systematically investigated and compared with C5TNa. Prior to evaluating the PTT efficacy of C5T‐ET in cells, its cellular uptake was assessed in 4T1 cells. As shown in Figure S13 (Supporting Information), both C5TNa and C5T‐ET entered the cells within 1 h, demonstrating that C5T‐ET can effectively internalize into tumor cells. Next, the in vitro phototherapeutic effect of C5TNa and C5T‐ET was assessed using a CCK‐8 assay. Under 808 nm light irradiation (0.6 W cm^−2^ for 5 min), the viability of 4T1 cells treated with C5T‐ET decreased significantly compared to those treated with C5TNa, underscoring the superior PTT efficacy of C5T‐ET (Figure S14A,B, Supporting Information). To investigate the mechanism of photoinduced cytotoxicity, flow cytometry using the Annexin V‐FITC/PI kit was performed to evaluate the apoptotic and necrotic pathways. As shown in Figure S14C (Supporting Information), phototherapy treatment induced 58.8% apoptosis and 3.4% necrosis in 4T1 cells. These results demonstrate that phototherapy mediated by C5T‐ET under 808 nm laser irradiation predominantly induces apoptosis rather than necrosis in 4T1 cells.

**Figure 7:**
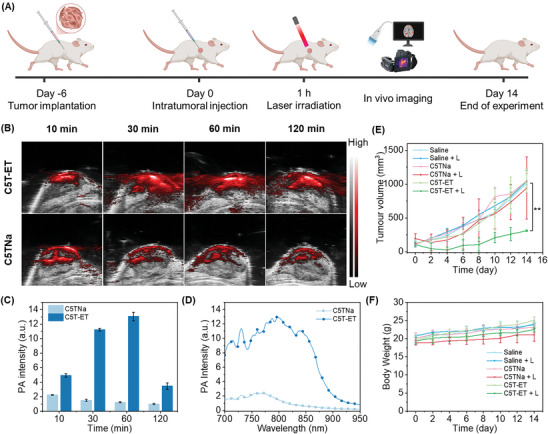
A) Schematic illustration of PAI in vivo with PTA. Figure created in https://BioRender.com (B) Overlaid PA and ultrasound images showing temporal changes in PA signals (red) following direct injection of C5T‐ET and C5TNa at the tumor site. PA signals were collected at 820 nm (For C5T‐ET) and 760 nm (For C5TNa) photoexcitation. Note: Grey and color bars indicate ultrasound and PA intensity, respectively. C) Time‐dependent (10–120 min) PA signals at tumor sites following the intratumoral (i.t.) injection of C5T‐ET and C5TNa, respectively. D) PA signal spectru following i.t. injection of C5T‐ET (at 60 min) and C5TNa (at 10 min). E) Tumor growth curves for various groups after different treatments, data are presented as mean±SD (n = 3 mice). ^**^ *P* < 0.01, Statistical significance was determined using ANOVA. F) Average body weight curves of mice in different treatment groups, data are presented as mean±SD (n = 3 mice).

The potential of C5T‐ET for in vivo PAI was investigated (**Figure** **7**A). Prior to PS injection (0 h), only a faint PA signal was detected, primarily attributed to deoxygenated and oxyhemoglobin (Figure S15, Supporting Information). Subsequently, 100 µL of 200 µm C5T‐ET was intratumorally injected (i.t.) into 4T1 tumor‐bearing BALB/c mice, and PA images of the tumors were captured at different time points (Figure 7B,C). The PA signal in the C5T‐ET‐treated mouse was significantly stronger, over 6.0 times that of the C5TNa‐treated mouse. This result aligns with the in vitro experiments, further validating the superior PA effect of C5T‐ET, even within the complex tumor microenvironment. The PA intensity was quantified at different time points (10, 30, 60, and 120 min) after injection. As illustrated in Figure 7C, the PA signals in the C5T‐ET‐treated group reached their peak at 60 min post‐injection. Interestingly, the mice treated with C5TNa exhibited the strongest PA signal at the initial time point (10 min), but this signal progressively decreased over time, likely due to its poor light stability. Meanwhile, the maximum PA signal of C5T‐ET inside the tumor was enhanced 6.1‐fold compared to the maximum signal of C5TNa (Figure 7D). Additionally, 3D‐rendered images of the NIR PA signals in the tumor tissues were generated (Figure S16, Supporting Information). These results demonstrated that C5T‐ET is suitable for in vivo PAI of tumors, providing precise guidance for laser irradiation during PTT.

Once C5T‐ET accumulation at the tumor site reached its peak, 808 nm laser irradiation (0.6 W cm^−2^) was applied to the tumor in 4T1 tumor‐bearing mice. The real‐time tumor temperature was monitored using an FLIR thermal camera. As shown in Figures S17 and S18 (Supporting Information), a rapid increase in temperature was observed in the tumor of the C5T‐ET‐treated group after 10 min of light irradiation, with the tumor temperature reaching 48.0 °C. In contrast, the mice treated with C5TNa and saline exhibited minimal temperature changes after irradiation. These results suggest that C5T‐ET can rapidly heat up at the tumor under NIR laser irradiation, demonstrating its potential for highly effective PTT in vivo.

Encouraged by the rapid temperature rise of C5T‐ET at the tumor, we evaluated the PTT efficacy in vivo by supervising the relative tumor volumes of 4T1 tumor‐bearing mice. Tumors in mice treated with C5TNa or subjected to 808 nm laser irradiation alone grew at the same rate as those in saline‐treated mice (Figure 7E), indicating that these treatments had negligible effects on tumor growth inhibition. However, with 808 nm laser irradiation (0.6 W cm^−2^) for 10 min, C5T‐ET effectively suppressed tumor growth. The remarkable antitumor efficacy of C5T‐ET was further confirmed by representative tumor images (Figure S19, Supporting Information) and hematoxylin and eosin (H&E) staining of tumor slices (Figure S20, Supporting Information). Moreover, higher levels of cell apoptosis, as assessed by terminal deoxynucleotidyl transferase dUTP nick end labeling (TUNEL), were observed in the C5T‐ET + Light group mice at the cellular level, further demonstrating its superior PTT effects compared to C5TNa (Figure S20, Supporting Information). This superior PTT outcome can be attributed to the high PCE of C5T‐ET, which allows for the rapid generation of sufficient heat at the tumor site to effectively ablation cancer cells.

Additionally, potential in vivo toxicity or side effects are critical considerations for PSs used in medical applications. Throughout the PTT treatment period, the negligible body weight loss (Figure 7F) suggests low in vivo toxicity and minimal side effects for all groups. Furthermore, at the end of the PTT treatment, no obvious histopathological abnormalities or signs of organ damage were examined in the major organs (Figure S21, Supporting Information), further confirming the excellent biosafety and minimal side effects of C5T‐ET.

## 3 Conclusion 

In summary, NIR‐absorbing organic PTAs based on cationic/anionic cyanine dye complexes can be effectively constructed through supramolecular assembly. Due to the strong CT interaction between the cationic and anionic Cys, the C5T‐ET complex exhibits a broad absorption profile extending beyond 800 nm, forming CT‐J aggregates with excellent NIR absorption capability. Notably, the nanoassembled C5T‐ET exhibits significantly enhanced PA signal intensity and photothermal effects compared to the pure monomer. Theoretical calculations and single‐crystal structure analysis revealed that the molecular pairs undergo a strong CT process and intermolecular aggregation, which contributes to the unique properties of the complexs and its high PCE of over 66%. More strikingly, fs‐TA results clearly demonstrate that the ultrafast internal conversion (IC) channel (22 ps) in C5T‐ET not only accounts for its superior photothermal performance compared to pure C5TNa but also provides valuable insight into the design of advanced PA materials. Furthermore, C5T‐ET facilitates high‐contrast tumor visualization via PAI and enables effective light‐induced thermal ablation of tumors in vivo.

## Conflict of Interest

The authors declare no conflict of interest.

## Ethical Approval

All animal experimental procedures were approved by the Animal Ethical and Welfare Committee of Shenzhen University (AEWC‐SZU) (Approval No.: IACUC‐202400029) and performed in accordance with all relevant policies and regulations.

## Supporting information



Supporting Information

## Data Availability

The data that support the findings of this study are available in the supplementary material of this article.
