# Supplementary material for: Electrostatic Co‐Assembly of Cyanine Pair for Augmented Photoacoustic Imaging and Photothermal Therapy
Source: Adv Sci (Weinh). 2025 Feb 14;12(13):2416905. doi: 10.1002/advs.202416905 (PMC11967836; doi:10.1002/advs.202416905)
Supplement: Supplementary file 1 — Supporting Information [file ADVS-12-2416905-s001.docx]

Supporting Information

Electrostatic Co-Assembly of Cyanine Pair for Augmented Photoacoustic Imaging and Photothermal Therapy

Haiqiao Huang, Yingnan Wu, Xin He, Yahang Liu, Jing-Hui Zhu, Mingrui Gu, Danhong Zhou, Saran Long, Yahui Chen, Lei Wang, Mingle Li *, Xiaoqiang Chen * and Xiaojun Peng *

H. Huang, Y. Wu, X. He, Y. Liu, J.H. Zhu, M. Gu,Y. Chen, L. Wang, M. Li, X. Chen, X. Peng

College of Materials Science and Engineering, Shenzhen University, Shenzhen 518060, P. R. China.

E-mail: ([limingle@szu.edu.cn](mailto:limingle@szu.edu.cn), chenxq@szu.edu.cn, [pengxj@dlut.edu.cn](mailto:pengxj@dlut.edu.cn))

H. Huang, Y. Wu, J.H. Zhu

College of Physics and Optoelectronic Engineering, Shenzhen University, Shenzhen 518060, P. R. China.

H. Huang, J.H. Zhu, X. Chen

Marshall Laboratory of Biomedical Engineering, Shenzhen University, Shenzhen 518060, P. R. China.

D. Zhou, S. Long, X. Peng

State Key Laboratory of Fine Chemicals, Dalian University of Technology, Dalian 116024, P. R. China.

Table of Contents

[Table of Contents 2](#_Toc190282704)

[General Experimental Details 3](#_Toc190282705)

[Synthetic method 9](#_Toc190282706)

[Figure S1-S24 11](#_Toc190282707)

[References: 25](#_Toc190282708)

General Experimental Details

**Material and sample preparation**

All solvents and reagents used were reagent grade. All reactions were performed in a nitrogen atmosphere with dry, freshly distilled solvents under anhydrous conditions. Silica gel (100-200 mesh) which obtained from Qingdao Ocean Chemicals was used for flash column chromatography for purification. Mass spectrometric data were obtained using LTQ Orbit rap XL instruments. The ^1^H NMR and ^13^C NMR spectra were recorded on a Bruker Avance II 400 MHz and 500 MHz spectrometer. The diameter of the nanoparticles was determined by dynamic light scattering (DLS) on a Zetasizer Nano (Malvem Zetasizer Nano ZS). Transmission electron microscopy (TEM) images were measured on a field emission transmission electron microscope (JEOL F200). Chemical shifts (*δ*) were reported as ppm in methanol-d4 with TMS as the internal standard. Bovine Serum Albumin (BSA) was purchased from Shanghai Sangon Biotech Co., Ltd. **ICG** was obtained from Shanghai Bide Pharmatech Ltd.. Water used in all experiments was doubly purified by Milli-Q Academic A10 Ultrapure water system equipment.

The solutions of **Cy5s**, **C5TNa** and **ICG** were typically prepared from 5.0 mM stock solutions in DMSO.

**Preparation of C5T-ET**

**C5T-ET** was directly mixes Cy-Et and C5TNa in equal molar ratio to form 5 mM stock solutions in DMSO.

**Measurements of absorption and fluorescence**

Absorption spectra were measured on a Shimazu Ultraviolet Visible Spectrophotometer UV-2600i (Shimazu). Fluorescence spectra were obtained with a VAEIAN CARY Eclipse fluorescence spectrophotometer (Serial No. FL0812:M018). The fluorescence quantum yield (*Φ*_f_) was measured with A Quantaurus-QY spectrometer (Hamamatsu, C11347).

**Single-Crystal X-Ray diffraction analysis**

Suitable single crystals of **C5TNa** and **C5T-ET** were selected from ether/methanol solution and covered with paraffin liquid. Single-crystal X-ray diffraction experiments were carried out on a Bruker D8 VENTURE diffractometer equipped with a CMOS detector using graphite-monochromated Mo Kα radiation (λ = 0.71073 Å). APEX-Ⅲ program 5 was used to determine the unit-cell parameters. The data were integrated with the SAINT program and were corrected for the Lorentz factor and polarization effects. Multi-scan absorption corrections were applied using SADABS. The molecular structures were solved by direct methods and refined by the full-matrix least squares on F2 using SHELXTL program (version 2014/7). All non-hydrogen atoms were refined anisotropically. The crystallographic data for this paper is available: CCDC-2407640 (for **C5TNa**), CCDC-2407641 (for **C5T-ET**) contain the supplementary crystallographic data for this paper. These data can be obtained free of charge from the Cambridge Crystallographic Data Centre via: www.ccdc.cam.ac.uk

**Photothermal Heating Detection** ^[1]^

The temperature of the water solutions treated with **C5TNa**, **C5T-ET** and **ICG** under 808 nm light (0.1, 0.3, 0.6, W cm^-2^, 10 min) was measured using an IR-thermal camera. The concentrations of samples used were 0, 10.0, 20.0, 40.0 μM for **C5T-ET**. The temperature was recorded every 30 s. The photothermal conversion efficiencies (η) were measured using a commonly described method:

$$\eta= \frac{hs\left( T_{\mathrm{Max}} - T_{\mathrm{Surr}} \right)-Q_{\mathrm{Dis}}}{I(1 - {10}^{-A})}$$

*h* is the heat transfer coefficient, *s* is the surface area of the container, *Q_Dis_* represents heat dissipated from the laser mediated by the solvent and container. *I* is the laser power and *A* is the absorbance at 808 nm.

$$hs=\frac{mC}{\tau_{s}}$$

*m* is the mass of the solution containing the photoactive material, *C* is the specific heat capacity of the solution (C_water_ = 4.20 J/(g•°C)), and *τ*_s_ is the associated time constant.

$$t= -\tau_{s}\ln\theta$$

*θ* is a dimensionless parameter, known as the driving force temperature.

$$\theta= \frac{T-T_{Surr}}{T_{Max}-T_{Surr}}$$

*T_Max_* and *T_Surr_* are the maximum steady state temperature and the environmental temperature, respectively.

**PA imaging**

PA imaging in vitro was executed using the Visualsonics LAZR Vevo system (Fujifilm, Japan) equipped with a 256-element transducer array (MX550D, 25 to 55 MHz) and a nanosecond pulsed laser (pulse width of 10 ns and repetition frequency of 20 Hz). Solutions of C5Tna, C5T-ET or ICG were enclosed within transparent polyurethane tubing and submerged in a water tank for acoustic coupling.

**Fluorescence lifetime measurements**

Time resolved fluorescence measurements were performed on freshly prepared samples using the time-correlated single photon counting (TCSPC) method (PicoQuant PicoHarp 300) at room temperature. Using deconvolution/fit program (PicoQuant FluFit), the time resolution was reached down to 10 ps. Emission was monitored at the wavelength of maximum fluorescence. Data analysis was performed with FluoFit software (Picoquant) using an exponential decay model. *c* = 10.0 μM.

**Femtosecond transient absorption (Fs-TA) spectra**

Femtosecond transient absorption (fs-TA) spectra and kinetics were measured using TA spectrometer (Helios fire, Ultrafast System). The fundamental pulses were generated with a Ti: sapphire laser system (Astrella, 800 nm, 100 fs, 7 mJ/pulse, and 1 kHz repetition rate, Coherent Inc.). A fraction of the fundamental beam was used to produce pump beams via an optical parametric amplifier (OPerA Solo, Coherent Inc.). White light continuum (WLC) probe beam was generated by focusing the fundamental beam into a CaF2 crystal and the time window limit is 8 ns.

**Quantum-chemical calculations**

The molecular geometries of C5T (-), Cy-ET (+), C5T-ET at the ground state and excited states were optimized using density functional theory (DFT) and time-dependent density functional theory (TD-DFT), respectively. In which the initial conformation of C5T-ET is from the crystal structure. Becke’s three-parameter hybrid exchange functions with Lee-Yang-Parr gradient corrected correlation functional (B3-LYP functional) was used, while B3LYP-D3 was used for C5T-ET dimer. The 6-311G(d) basis set was used and the solvent effect of water with SMD model was included in all calculations. The excited states were calculated using TD-DFT at the same theoretical level. All the quantum-chemical calculations and Electrostatic Potential Surface (ESP) were completed with the Gaussian 16 package.

**Cell and Culture Conditions**

4T1 cells were maintained in DMEM medium, all of them were supplemented with 1% penicillin streptomycin and 10% FBS, and atmosphere of 5% CO_2_ and 95% air at 37ºC. When used for imaging, all types of cells were cultured on 35 mm glass-bottom culture dishes for 12-24 h.

**Real-time cellular uptake of C5TNa or C5T-ET in 4T1 cells**

For real-time cellular uptake assays, 1 × 10^5^ 4T1 cells were firstly plated onto 35 mm confocal dishes and incubated for 24 h. Then, 2.0 μM **C5TNa or C5T-ET** was added to the cells, and the fluorescence images were captured at various time points (0.5, 1, 2, 3 hours). The corresponding fluorescence intensity was measured using confocal laser scanning microscopy (CLSM). The excitation wavelength was 635 nm, and collection wavelength was from 700 nm to 800 nm.

**Dark Cytotoxicity Evaluation**

The cytotoxicity effects of **C5TNa** and **C5T-ET** were evaluated by 4T1 cell using a MTT assay. The 4T1 cells were planted in 96 well cell culture plate at density of 1 × 10^4^ cells per well for 24 h at 37^o^C, 5% CO_2_. Then, **C5TNa** or **C5T-ET** with various concentrations (0, 0.62, 1.20, 2.50, 5.00, 10.00 μM for **C5TNa** and **C5T-ET**) were added and grown for 24 h. For the Cell Counting Kit-8 (CCK-8) assay, the medium and CCK-8 (Beyotime, C0039) reagent were prepared in a ratio of 10:1. The medium was removed, and the cells were washed with PBS and incubated with diluted CCK-8 solution at 37 °C for 2 h in the dark. Finally, the absorption of each well was recorded at 490 nm on a multi detection microplate reader (Thermo Fisher Scientific).

The cell viability was calculated by the following equation:

Cell viability (%) = (*OD*_PS_ − *OD*_blank_)/(*OD*_control_ − *OD*_blank_)×100

**Light Cytotoxicity Evaluation**

The 4T1 cells were planted in 96 well cell culture plate at density of 1 × 10^4^ cells per well for 24 h at 37^o^C, 5% CO_2_. Then, **PSs** with various concentrations (0, 2.50, 5.00,10.00 μM for **C5TNa** or **C5T-ET**) were added and further incubated for 2 h prior to irradiation with 808 nm light at 0.6 W cm^−2^ for 5 min. After an overnight incubation, The medium was removed, and the cells were washed with PBS and incubated with diluted CCK-8 solution at 37 °C for 2 h in the dark. Finally, the absorption of each well was recorded at 490 nm on a microplate reader (Thermo Fisher Scientific).

**Annexin V-FITC/propidium iodide (PI) Apoptosis Detection Kit**

Annexin V-FITC/propidium iodide (PI) Apoptosis Detection Kit (Beyotime, China) was used for detection of **C5T-ET** mediated photoinduced cell death. Briefly, 4T1 cells (1×10^6^ cells per well) were seeded in 6-well plates and cultured overnight. Then cells were incubated with different conditions and divided into six groups: (1) Control, (2) 808 nm light irradiation (0.6 W cm^-2^, 5 min), (3) incubated with **C5T-ET** (7.2 μM) without light (dark), (4) **C5T-ET** (7.2 μM) + 808 nm light (0.6 W cm^-2^, 5 min). After incubation for another 12 h, the cells were centrifuged at 1300 rpm for 5 min and resuspended in Annexin V binding buffer (10 mM HEPES, 150 mM NaCl, 5 mM KCl, 1 mM MgCl_2_, 1.8 mM CaCl_2_). Then cells were stained with Annexin V FITC/propidium iodide (PI) Apoptosis Detection Kit according to the manual. Finally, cells were analyzed on a flow cytometer.

***In vivo* experiments**

**Animals and Tumor Model**

All animal experimental procedures were approved by the Animal Ethical and Welfare Committee of Shenzhen University (AEWC-SZU) (Approval No.: IACUC-202400029) and performed in accordance with all relevant policies and regulations. Specific female Balb/c mice, 4-6 weeks of age, originally purchased from Guangdong Medical Laboratory Animal Center, were used to establish breast cancer mouse model. Briefly, 1×10^6^ 4T1 cells were injected subcutaneously into the selected positions to establish the breast tumor model of Balb/c mice. Tumors were allowed to grow to about 100 mm^3^ in volume before used for *in vivo* imaging and phototherapy.

**In *vivo* tumor imaging**

For *in vivo* tumor imaging, the 4T1 tumor-bearing Balb/c mice were intratumorally injected with **C5TNa or C5T-ET** (200 μM, 100 μL), and the *in vivo* PA signals were monitored at different post-injection time (0, 10, 30, 60, 120 mins for **C5TNa** or **C5T-ET**) by using the Visualsonics LAZR Vevo system (Fujifilm, Japan).

For *in vivo* tumor photothermal imaging, the 4T1 tumor-bearing Balb/c mice were divided three groups: 1) Injecting Saline (100 μL) into the mice; 2) intratumorally injected with **C5TNa** (200 μM, 100 μL). 3) Intratumorally injected with **C5T-ET** (200 μmol, 100 μL). The photothermal images were recorded by IR-thermal camera signals were monitored at irradiation (808 nm, 0.6 W cm^-2^) time (0, 1, 2, 3, 4, 5, 6, 7, 8, 9, 10 min) after 1 h post-injection.

***In* *vivo* Phototherapy Evaluation**

To confirm the *in vivo* Phototherapy efficacy of **C5T-ET**, all mice were divided into six groups (and each group contained 3 mice) and subjected to treatments: (1) treated with saline (as a control), (2) treated with saline and plus light (808 nm laser, 0.6 W cm^-2^, 10 min), (3) treated with only **C5TNa** (200 μM, 100 μL), (4) treated with **C5TNa** (200 μM, 100 μL) followed by light (808 nm laser, 0.6 W cm^−2^, 10 min), (5) treated with only **C5T-ET** (200 μM, 100 μL), and (6) treated with **C5T-ET** (200 μM, 100 μL) followed by light (808 nm laser, 0.6 W cm^-2^, 10 min). In the following 2 weeks, the tumor volume of all mice was measured every day using a vernier calliper. Then, the greatest longitudinal diameter (length) and the greatest transverse diameter (width) were used to calculate the tumor volume. Tumor volume = width × width × length/2. In addition, after 14-day post-treatment.

***In vivo* biosafety assay**

During the course of treatments, the body weights of all mice were monitored every other day. 14 days later, the mice were euthanized, and main organs including heart, liver, spleen, lung, kidneys were harvested for histological analysis by means of hematoxylin-eosin (H&E) staining.

**Statistical analyses**

The data were analyzed by one-way analysis of variance using SPSS (version 25.0; IBM Corp., Armonk, NY, USA). Tukey's post hoc test was used to determine the significance of all pairwise comparisons of interest. *p*<0.05 was considered to indicate a statistically significant difference.

Synthetic method

1. **Synthesis routes of C5TNa, Cy5s and Cy7**

**Scheme S1**. Synthetic route and chemical structures of seven pentamethine cyanine dyes. and Cy7.

According to the literature methods, **Cy5s** were prepared. ^[2,3]^ Cy7-Cl-Et was synthesized according to our previous work. ^[4]^

**1.1**  **General synthesis method of the** **intermediate** **3a-f**

2,3,3-trimethyl-3H-indole 1 (3.98 g, 25.0 mmol, 1 eq) and corresponding haloalkane 2a-f (50.0 mmol, 2 eq) were added into a 100 mL stand-up round bottom flask containing 50 mL toluene under nitrogen. The mixture was then heated to reflux the reaction overnight. Subsequently, after the reaction was terminated and cooled down to room temperature, 100 mL ether was added to allow precipitation. The resulting solid precipitate was then filtered, washed with ether, and dried so that solid powder 3a-f was obtained. The final product was used directly in the dye synthesis without further purification.

**1.2 General synthesis method of Cy5 a-f**

2,3,3-trimethyindolenium iodide salt 3a-f (4.4 mmol, 2.2 eq), compound 4 (0.60 g, 2.0 mmol, 1 eq), and anhydrous NaOAc (0.37 g, 4.4 mmol, 2.2 eq) were mixed with 20 mL ethanol and heated to 82°C for 2 h under nitrogen. The reaction solution was then cooled down and poured into 100 mL saturated sodium iodide, and was subsequently extracted by DCM (3×50 mL), dried over anhydrous Na_2_SO_4_, and evaporated in a vacuum. The residue was purified using chromatography (silica gel), in which 80-40:1 DCM/methanol (v/v) was used as the eluting solvent. After the solvent was removed, the solid product was obtained Cy5s.

**1.3 Synthesis of C5TNa.**

Compound 5 (4.4 mmol, 2.2 eq), compound 4 (0.52 g, 2.0 mmol, 1.0 eq), and anhydrous NaOAc (0.37 g, 4.4 mmol, 2.2 eq) were mixed with 20 mL acetic anhydride and heated to 110°C for 2 h under nitrogen. The reaction solution was then cooled down and poured into 100 mL saturated brine, and was subsequently extracted by DCM (3×50 mL), dried over anhydrous Na_2_SO_4_, and evaporated in a vacuum. The residue was purified using chromatography (silica gel), in which 80-40:1 DCM/methanol (v/v) was used as the eluting solvent. After the solvent was removed, the solid product was obtained C5TNa (0.47 g, yield 52%).

^1^H NMR (400 MHz, Acetone-d6) *δ* 7.81 (t, *J* = 13.2 Hz, 2H), 6.45 (t, *J* = 12.5 Hz, 1H), 6.09 (d, *J* = 13.8 Hz, 2H), 2.06 (s, 12H).

^13^C NMR (101 MHz, Acetone-d6) *δ* 206.34, 177.50, 169.72, 150.07, 126.10, 115.81, 115.13, 109.45, 95.70, 27.09.

ESI-HRMS (*m*/*z*): calcd for: C_25_H_17_N_6_O_2_^–^ [M]^–^, 433.1418; found, 433.1418.

Figure S1-S24

**Table S1.** The optical properties and biological application of recently reported BODIPY or Cys J-aggregates.

| Dye | Design strategies | Absorption wavelength  (nm) | | application | References |
| --- | --- | --- | --- | --- | --- |
|  |  | Monomeric | J-aggregate |  |  |
|  | Electrostatic co-assembly of cyanine pair | 738 | 790 | PAI  and PTT (PCE = 66%) | This Work |
|  | Inside hollow mesoporous silica nanoparticles | 826 | 965  1040 | Shortwave infrared imaging | *J. Am. Chem. Soc.* **2019**, *141, 32*, 12475–12480 |
| 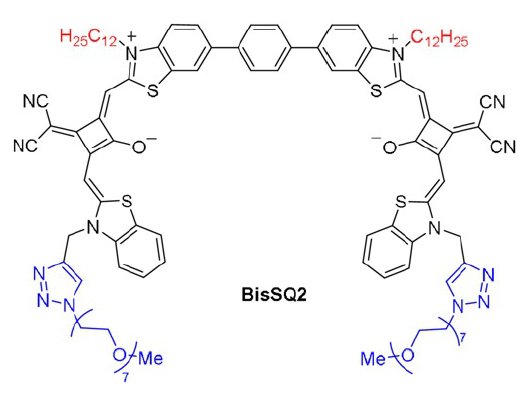 | Covalently linked by a para-phenylene spacer | 726 | 780 | Bulk heterojunction solar cells | *Angew. Chem. Int. Ed.* **2021**, *60*, 11949-11958. |
|  | Negatively charge in microenvironments | 630 | 700 | PDT | *Biomaterials* **2021**, *269*, 120532. |
| 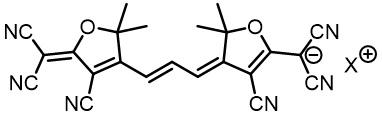 | Counterion engineering | 647 | 668 | PDT | *Angew. Chem. Int. Ed.* **2022**, *61*, e202203093. |
|  | NaCl treatment | 766 | 910 | PTT  (PCE = 57.59%) | *Angew. Chem. Int. Ed.* **2023**, *62*, e202216109. |
|  | Introducing a branched structure | 878 | 1007 | PAI and fluorescence bioimaging | *Angew. Chem. Int. Ed.* **2024**, *63*, e202406694. |
|  | Atom-programming strategy | 677 | 783 | PTT (PCE = 64.3%) | *ACS Nano* **2021**, *15, 3*, 5032–5042 |
|  | Atom-programming strategy | 673 | 790 | PTT (PCE = 59.8%) | *Small* **2021**, *17*, 2101180. |
|  | Ethenylene-bridged | 1: 769  2: 1125 | 1: 873  2: 1350 | 2: PTT (PCE = 63%) | *Sci. Adv.* **2022**, *8*, eadd5660. |
|  | Glycosylated | 688 | 740 | PDT | *Angew. Chem. Int. Ed.* **2023**, *62*, e202309786. |
|  | Glycosylated | 756 | 840 | PTT (PCE = 55%) | *J. Colloid Interface Sci*. **2022**, *612*, 287. |
| 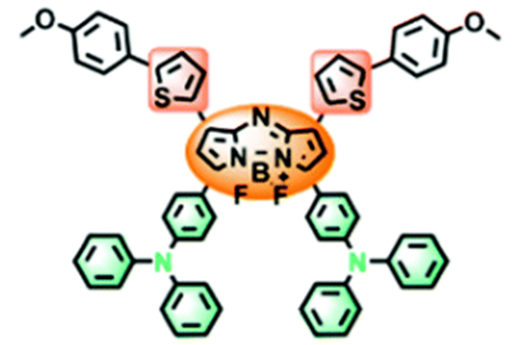 | Introducing steric hindrance | 843 | 939 | PTT (PCE = 35.6%) | *J. Mater. Chem. B* **2022**, *10*, 1650. |
| 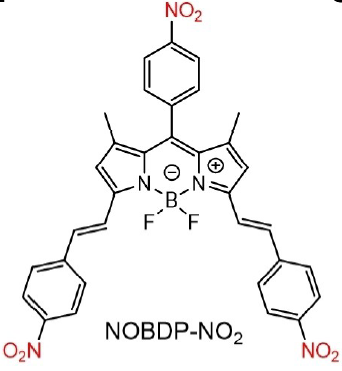 | Aromatic substituent strategy | 665 | 750 | Bioimaging and information encryption | *Angew. Chem. Int. Ed.* **2023**, *62*, e202313166. |
|  | Amphiphilic side chains | 694 | 707  799 | Near-Infrared luminescence | *Chem. Mat.* **2024**, *36*, 3745-3753. |


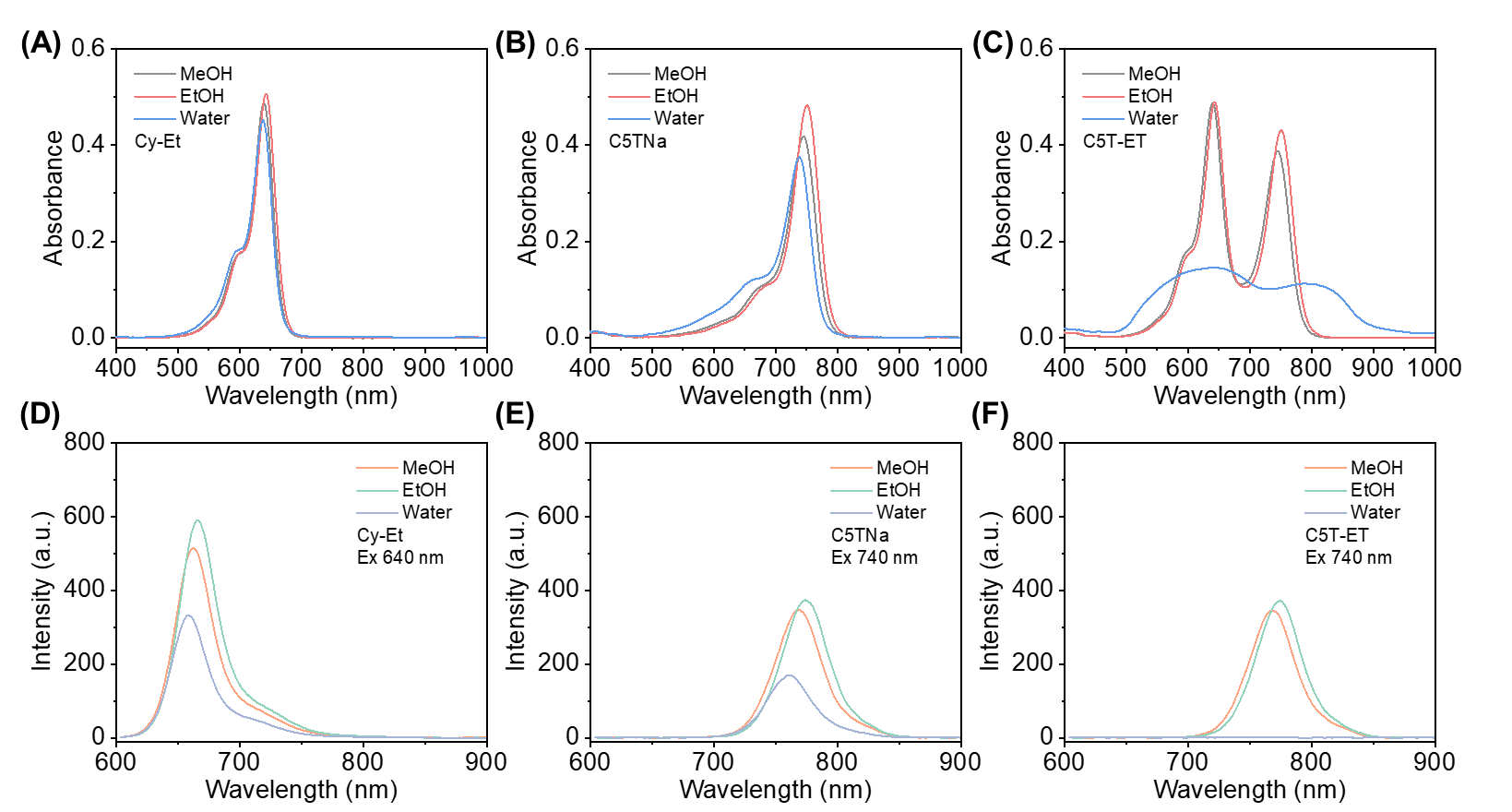


**Figure S1.** The absorption (A, B, C) and emission (D, E, F) spectra of Cy-Et, C5TNa and C5T-ET in methanol, ethanol and water, respectively. (c = 2.0 μM)


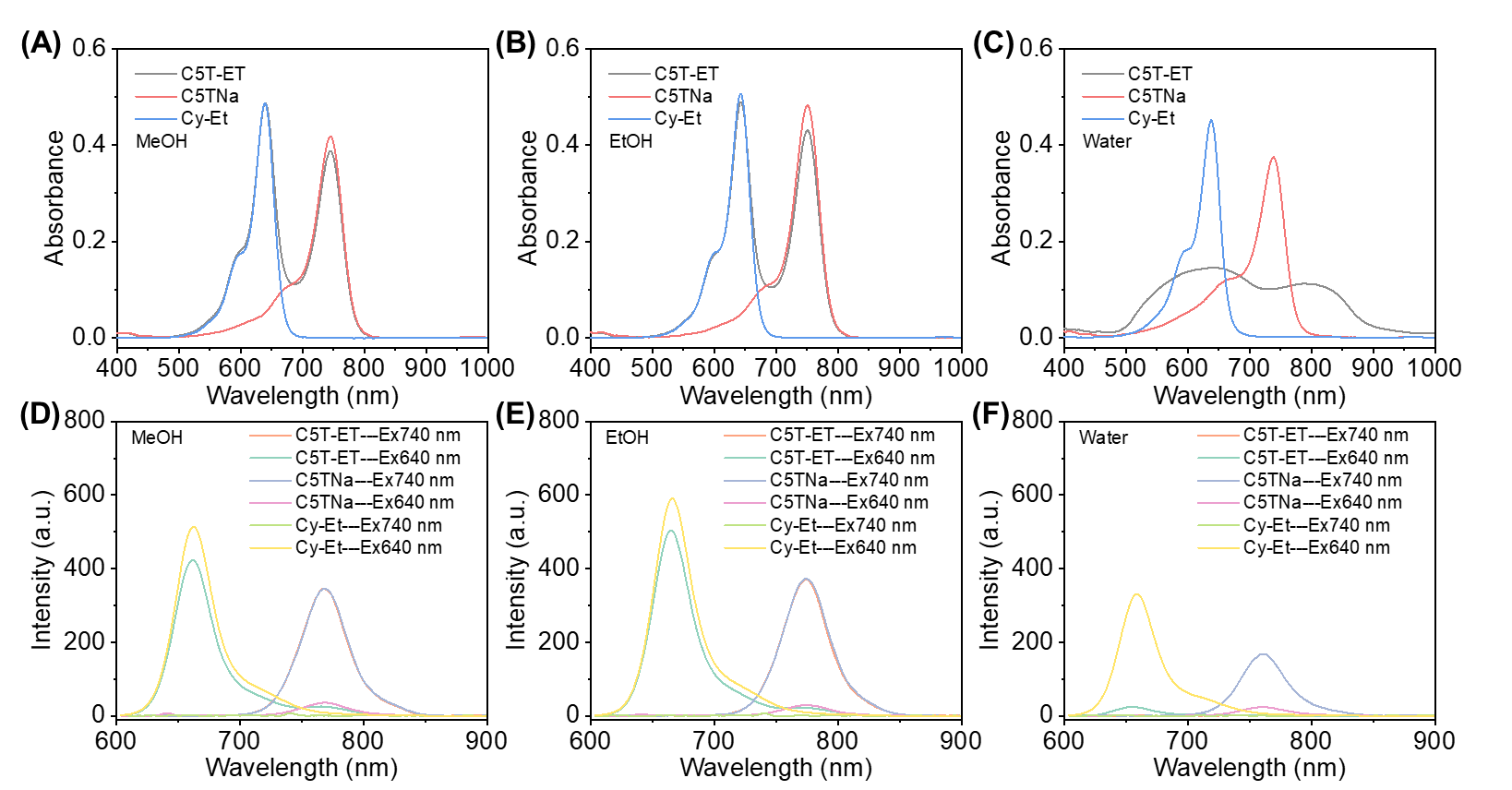


**Figure S2.** The absorption and emission spectra of Cy-Et, C5TNa and C5T-ET in methanol (MeOH), ethanol (EtOH) and water. (c = 2.0 μM)


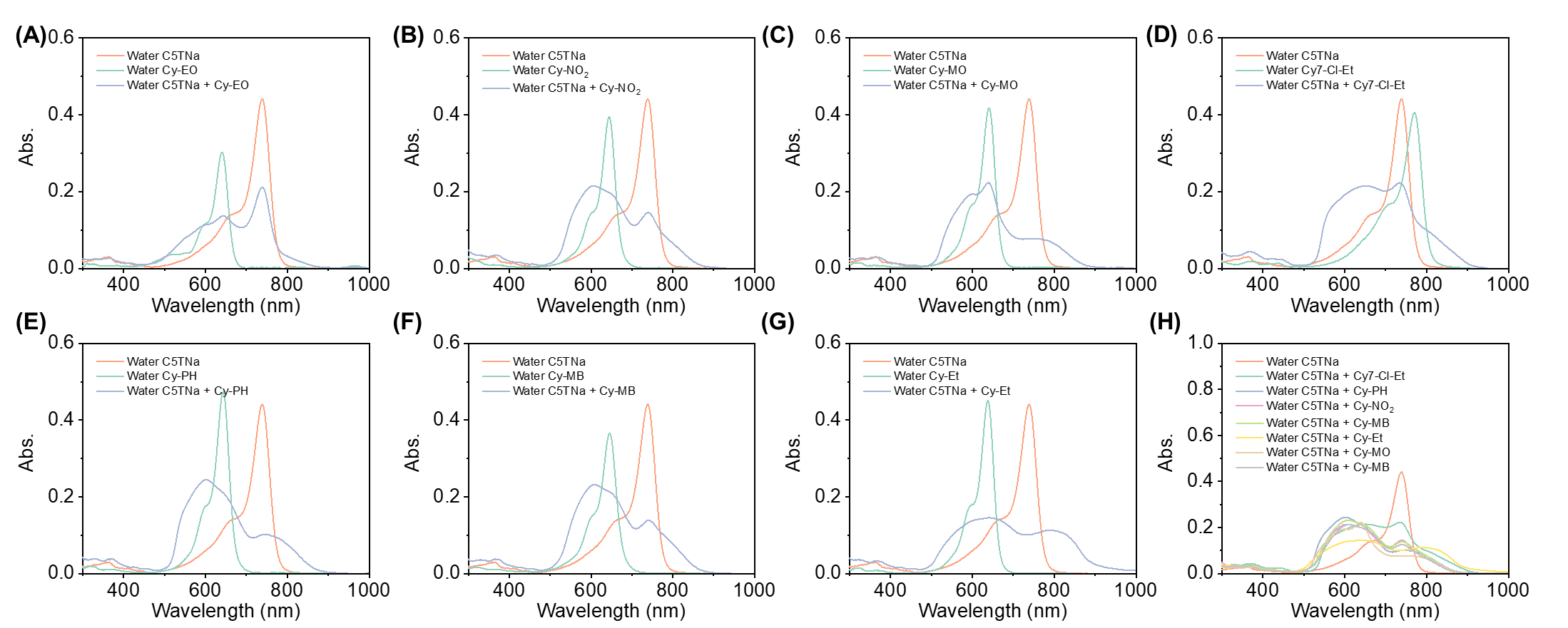


**Figure S3.** The absorption spectra of Cy5s or **Cy7-Cl-Et** (c = 2.0 μM) with C5TNa (c = 2.0 μM) in Water.

**Table S2.** The oil - water partition coefficient (Log*P*) of all t cyanine dyes and these the absorption of the mixed molecules at 808 nm.

| Dye | C5TNa | Cy7-Cl-Et | Cy-NO2 | Cy-PH | Cy-MB | Cy-EO | Cy-MO | Cy-Et |
| --- | --- | --- | --- | --- | --- | --- | --- | --- |
| LogP | 1.049 | 0.517 | 0.013 | 0.605 | 1.044 | -0.092 | 0.22 | 1.354 |
| (Abs.) at 808 nm | 0.005 | 0.094 | 0.058 | 0.068 | 0.069 | 0.028 | 0.059 | 0.111 |


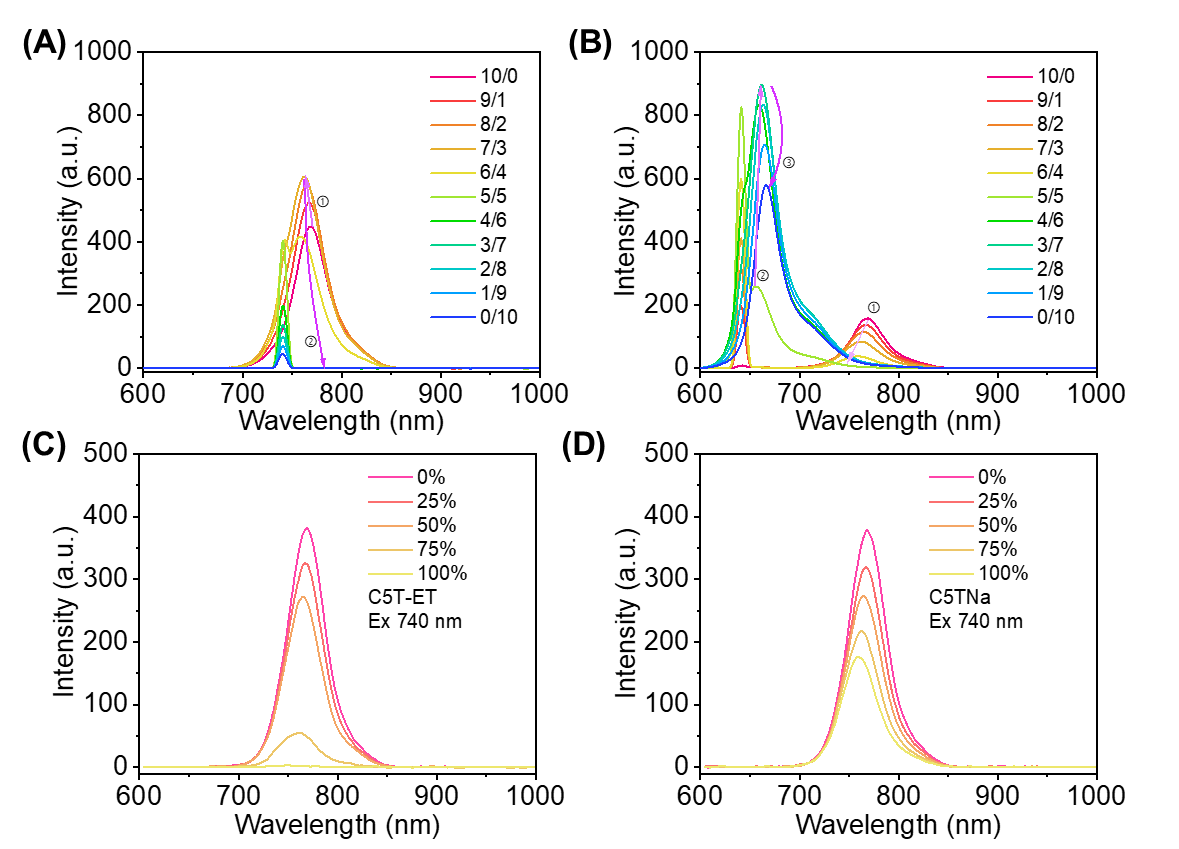


**Figure S4.** Emission spectra of C5TNa mixed with different molar ratios of Cy-Et in water. (The total concentration of C5TNa and Cy-Et was 10.0 μM. Legend: C5TNa / Cy-Et) (A) *λ*_ex_ = 740 nm, and (B) *λ*_ex_ = 640 nm. (C) Emission spectra of C5T-ET and (D) C5TNa in MeOH-H_2_O solutions with varied *f*_water_. c = 2.0 μM, *λ*_ex_ = 740 nm.


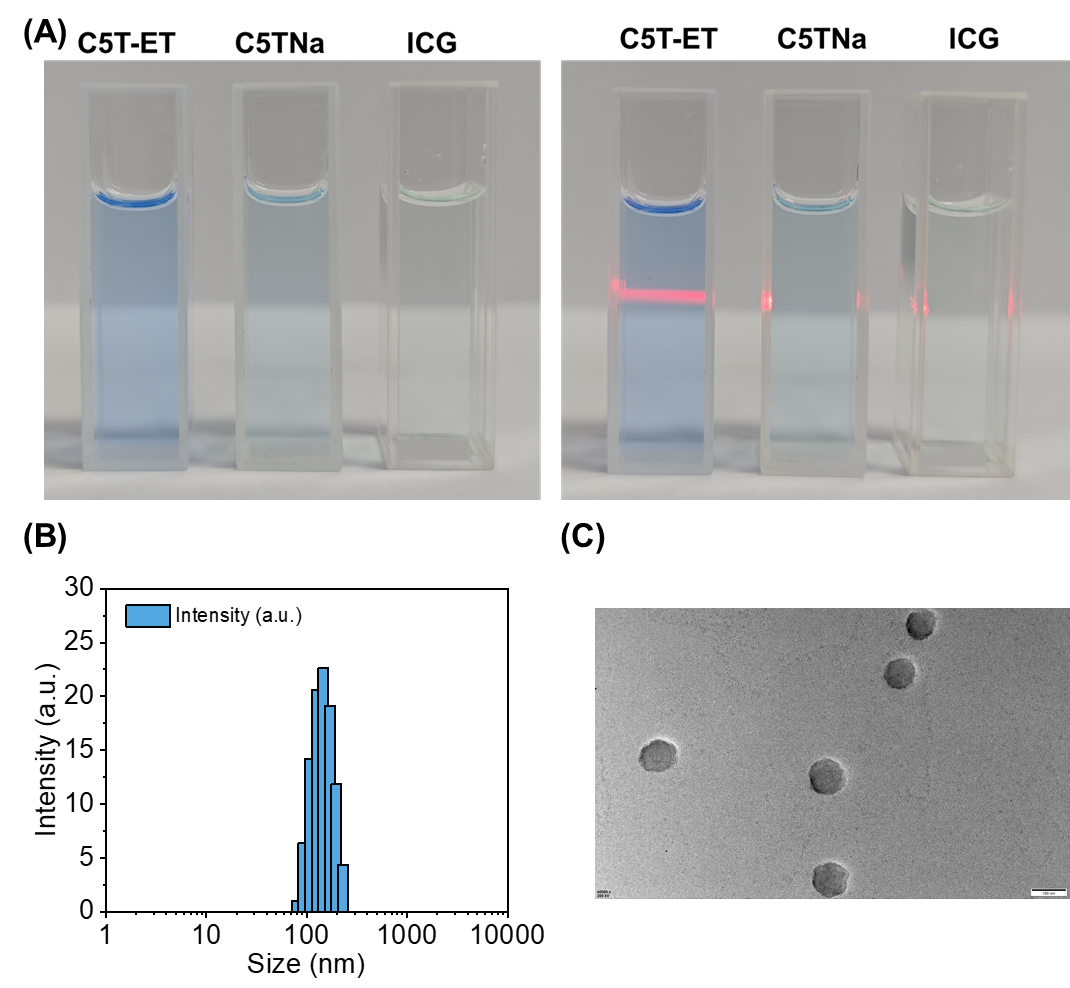


**Figure S5.** (A) Photographs of C5T-ET, C5TNa and ICG dissolved in water and illuminated by a red laser pointer. (B) DLS profile of C5T-ET. (C) TEM image of C5T-ET.


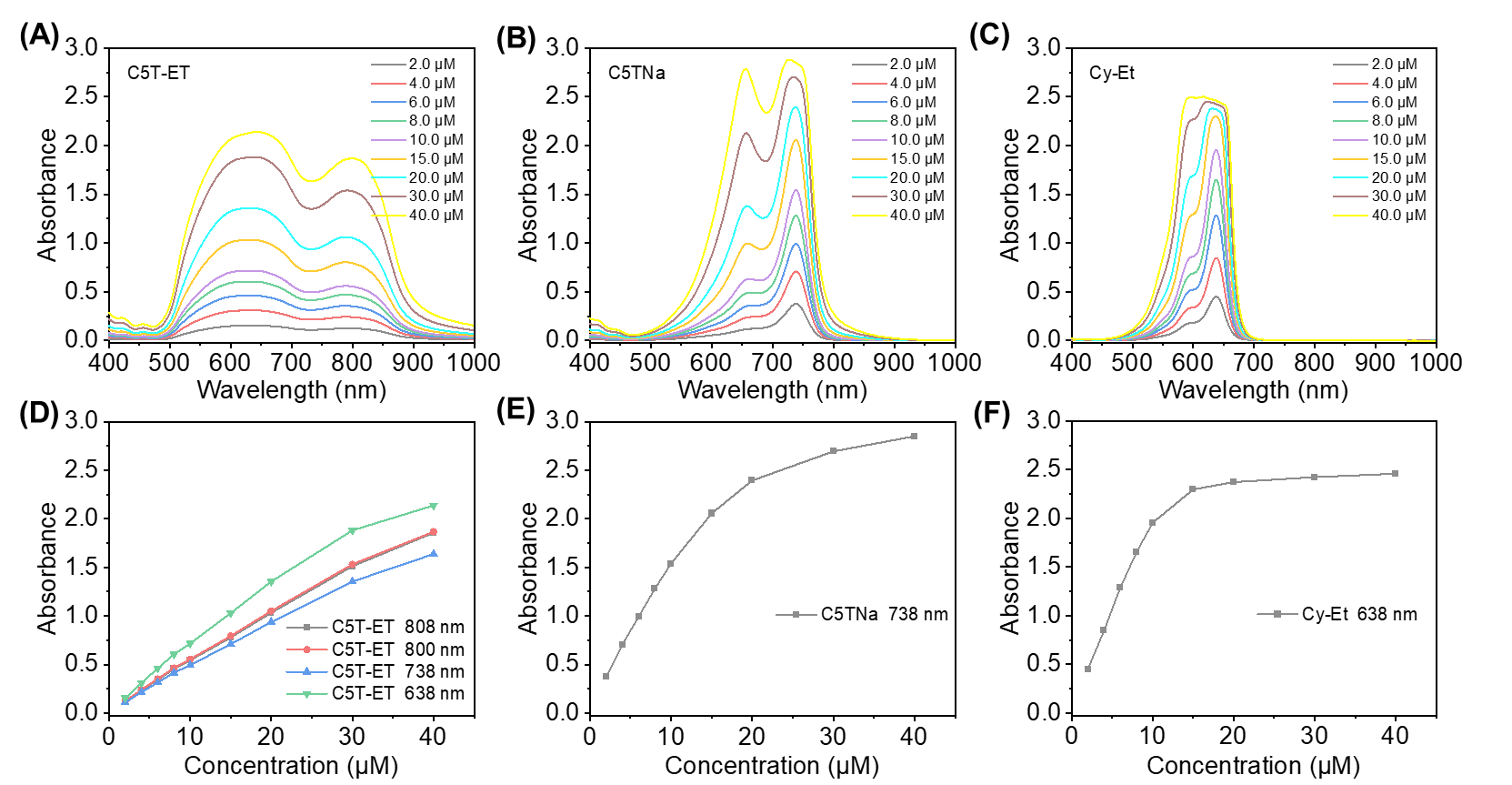


**Figure S6.** Absorption spectra of C5T-ET (A), C5TNa (B) and Cy-Et (C) in water at various concentrations (2.0 – 40.0 μM). The linear relationship observed in absorbance maximum wavelength versus concentration plots of C5T-ET (D), C5TNa (E) and Cy-Et (F) in water.


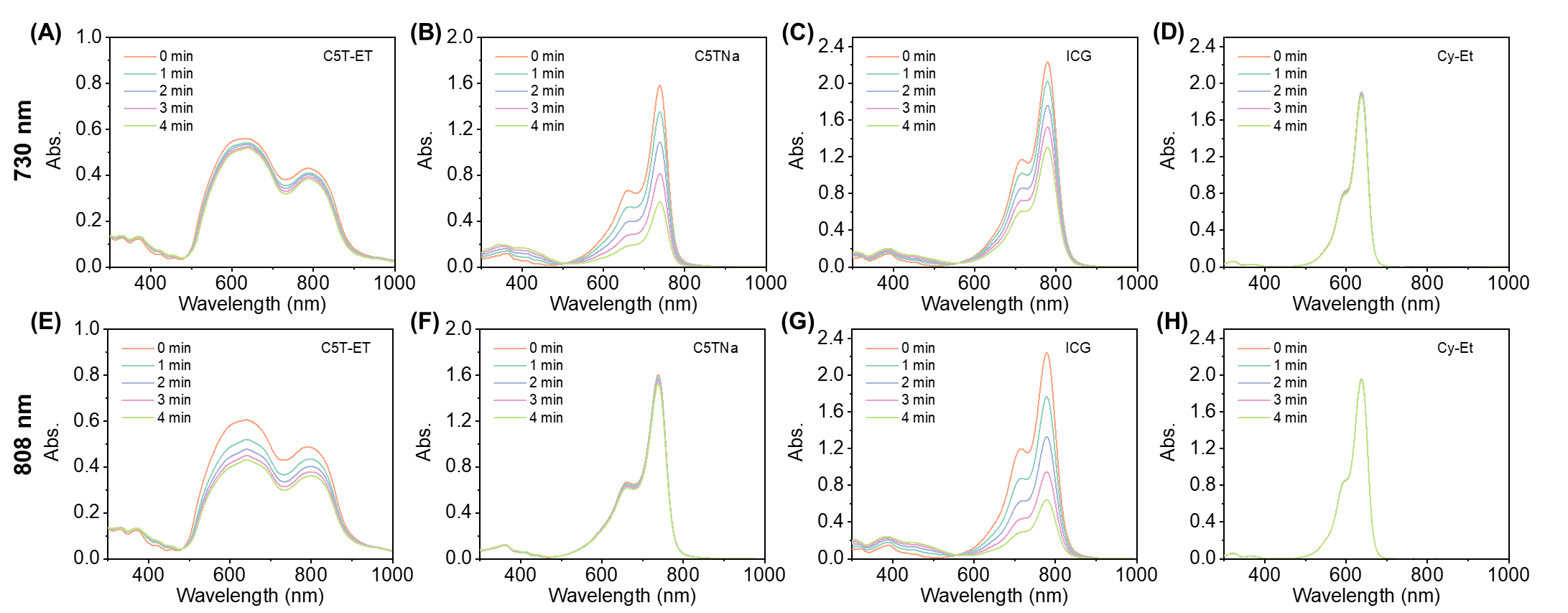


**Figure S7.** Exploration of improvement of cyanine’s photostability. The absorption changes of C5T-ET (A), C5TNa (B), ICG (C), and Cy-Et (D) under 730 nm laser irradiation with a power density of 0.1 W cm^−2^. The absorption changes of C5T-ET (E), C5TNa (F), ICG (G), and Cy-Et (H) under 808 nm laser irradiation with a power density of 0.1 W cm^−2^. (c = 10.0 μM)


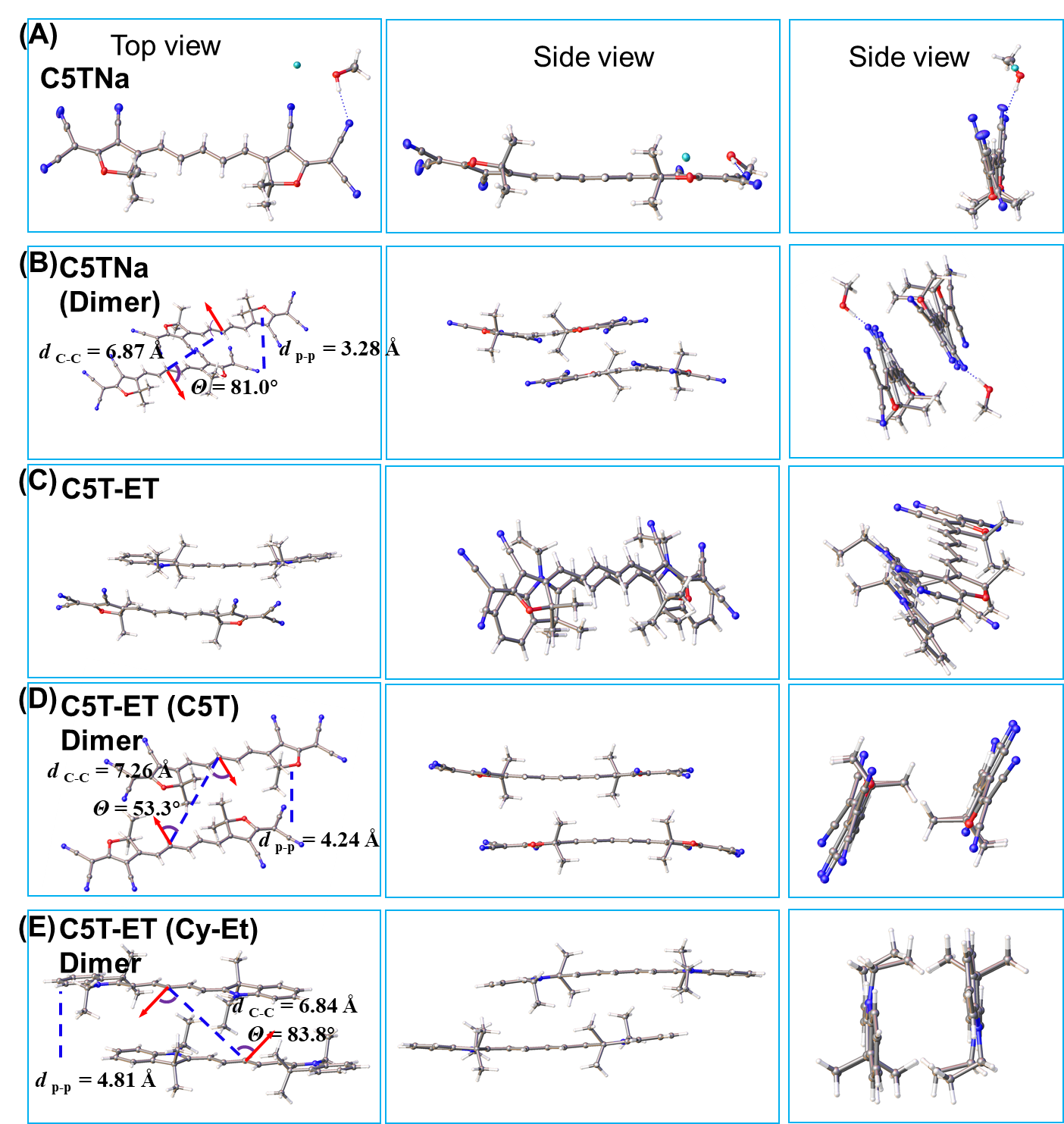


**Figure S8.** (A) Single-crystal structures of C5TNa in tilt view (top), side view (right) and side view (left). (B) Packing structures of C5TNa in dimers with transition dipoles of S1 denoted as red arrows in tilt view (top), side view (right) and side view (left). (C) Single-crystal structures of C5TNa. Packing motifs of aggregated dimers of (E) C5T-ET (part of C5T) and (F) C5T-ET (part of Cy-ET) with transition dipoles of S1 denoted as red arrows in tilt view (top), side view (right) and side view (left).


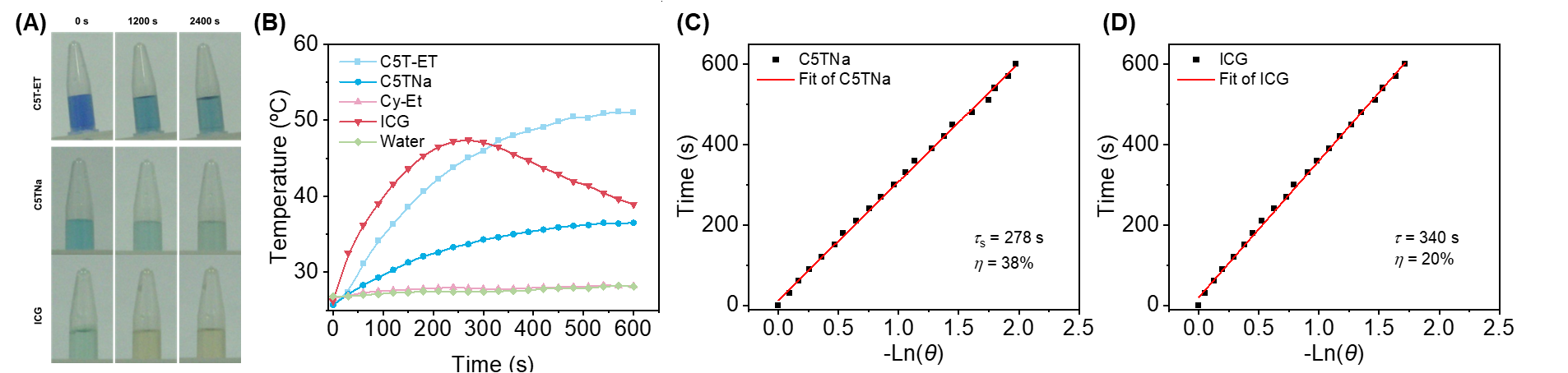


**Figure S9.**  (A) The photographs of different compound solutions before and after light irradiation. (B) Photothermal conversion of C5T-ET, C5TNa, Cy-Et, ICG (20.0 µM) and water under 808 nm laser irradiation (0.6 W cm^−2^). Heat-transferring time constant (𝜏_s_) determined by time−temperature data from the cooling period, and Photothermal conversion efficiency (𝜂) calculation of C5TNa (C) and ICG (D).


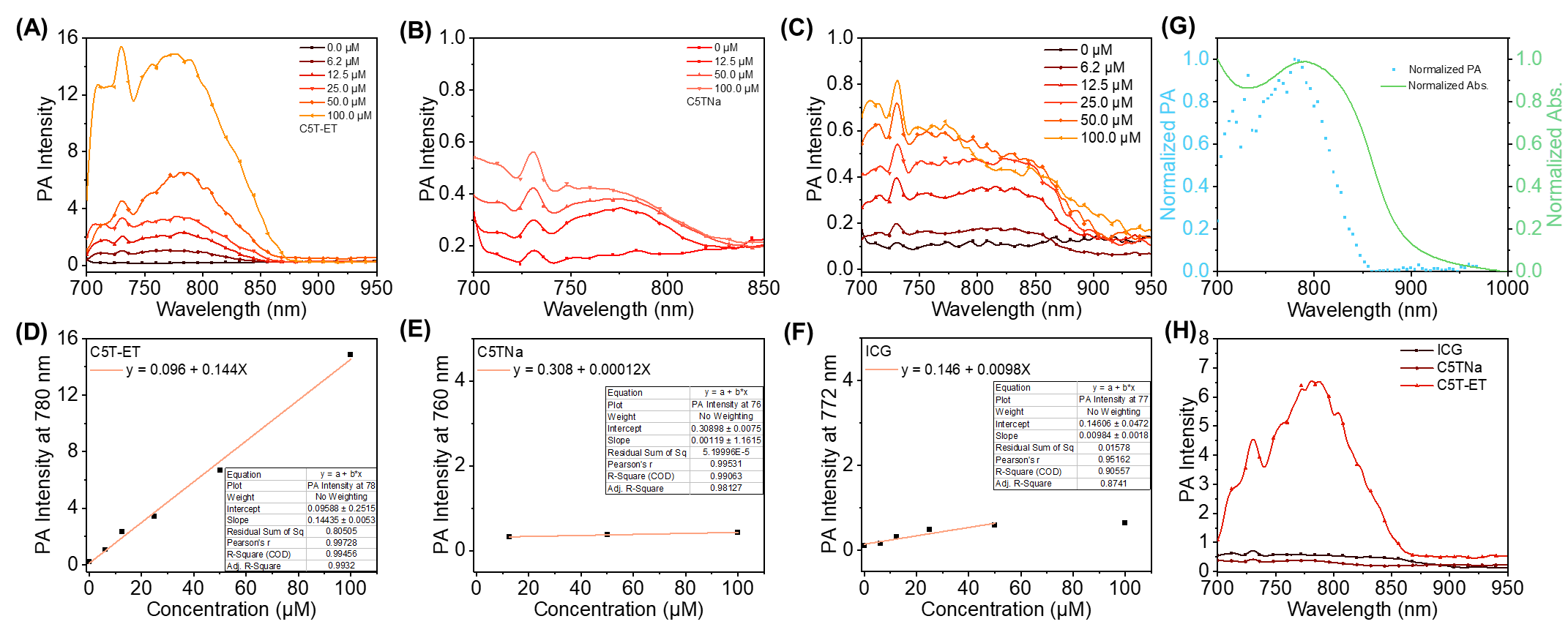


**Figure S10.** PA spectra of (A) C5T-ET, (B) C5TNa, and (C) ICG at different concentrations in water. PA intensity as a function of concentrations of (D) C5T-ET, (E) C5TNa, and (F) ICG. (G) Overlay of normalized absorbance and photoacoustic signal generation spectra for C5T-ET. (H) PA spectra of C5T-ET, C5TNa, and ICG at 50.0 µM in water.

**Table S3**  The photophysical properties of Cy-Et, C5T and C5T-ET calculated with TD-DFT method. ^[5]^

| **Dyes** | **Excited state** | ***λ*_Abs_** | ***f*_Abs_** | **Transition** | ***λ*_em_ (S1)** | ***f*_em_** |
| --- | --- | --- | --- | --- | --- | --- |
| C5T-ET | S1  S2  S3  S4 | 762  672  646  585 | 0.9875  1.1958  1.4839  1.3205 | H→L  H-1→L  H→L+1  H-1→L+1 | 990 | 0.1855 |
| C5T | S1 | 709 | 2.6679 | H→L | 726 | 2.6417 |
| Cy-Et | S1 | 612 | 2.3466 | H→L | 655 | 2.3372 |


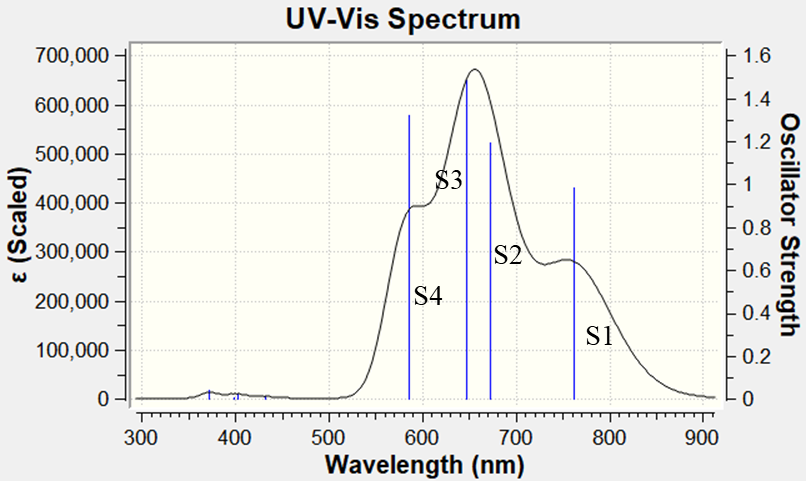


**Figure S11.** The absorption spectrum of C5T-ET based on TD-DFT calculations.

**Table S4** The excited state of C5T-ET was analyzed using Multiwfn.

|  | ***λ*_abs_/nm** | **Transition** | **Fragment** | **Hole%** | **Electron %** | **Overlap%** | **Type** |
| --- | --- | --- | --- | --- | --- | --- | --- |
| S1 | 762 | H→L | Cy-Et | 53.91 | 2.15 | 10.78 | CT (CyEt→C5T)  LE (C5T→C5T) |
|  |  |  | C5T | 46.09 | 97.85 | 67.16 |  |
| S2 | 672 | H-1→L | Cy-Et | 46.76 | 2.15 | 10.04 | LE (C5T→C5T)  CT (CyEt→C5T) |
|  |  |  | C5T | 53.24 | 97.85 | 72.18 |  |
| S3 | 646 | H→L+1 | Cy-Et | 53.91 | 97.56 | 72.52 | LE (CyEt→CyEt)  CT (C5T→CyEt) |
|  |  |  | C5T | 46.09 | 2.44 | 10.60 |  |
| S4 | 585 | H-1→L+1 | Cy-Et | 46.76 | 97.56 | 67.54 | CT (C5T→CyEt)  LE (CyEt→CyEt) |
|  |  |  | C5T | 53.24 | 2.44 | 11.39 |  |


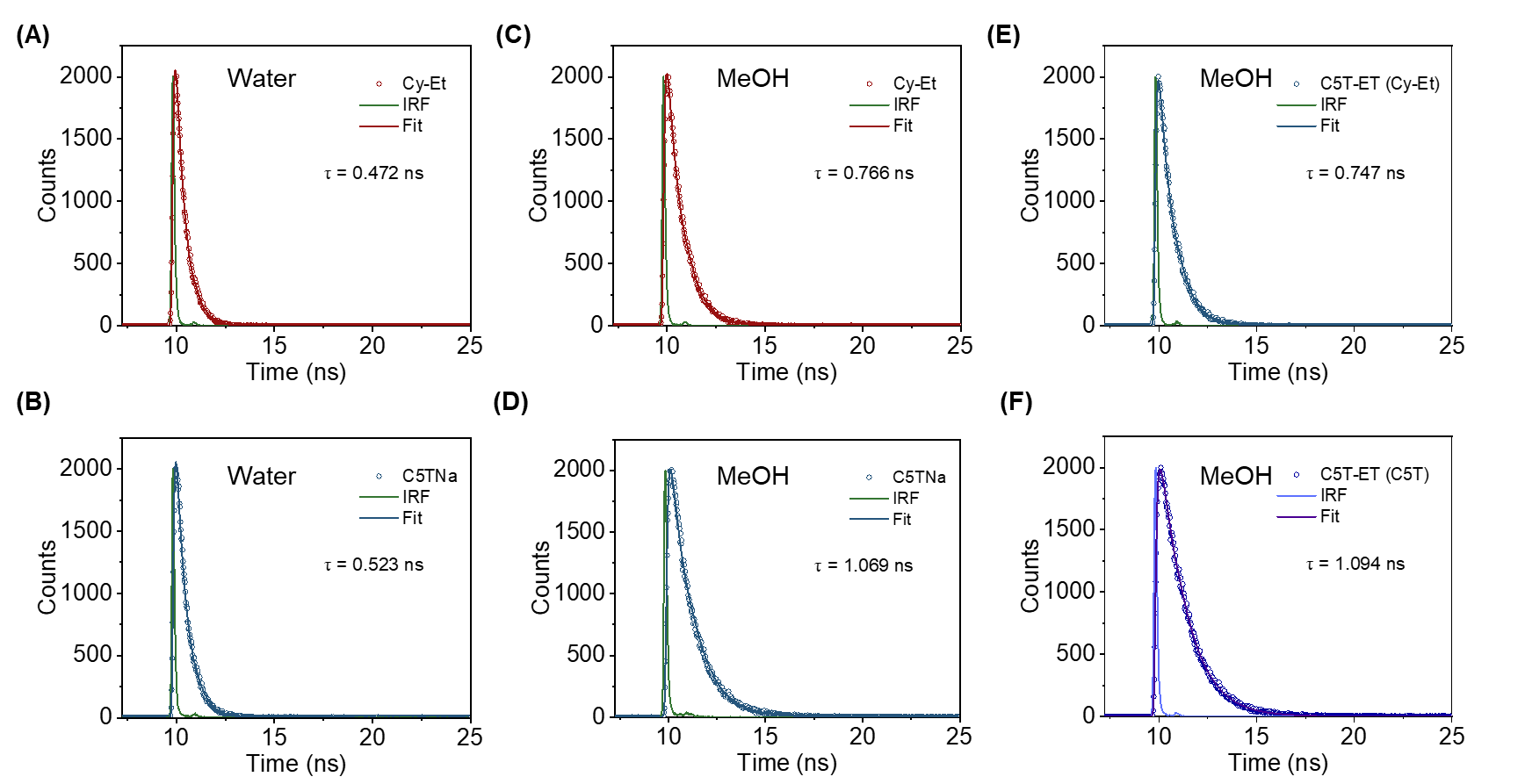


**Figure S12.** Fluorescence lifetime decay curve of the dyes in water, 10.0 μM. (A) Cy-Et in water, *λ*_ex_ = 629 nm, *λ*_detection_ = 656 nm; (B) C5TNa in water, *λ*_ex_ = 723 nm, *λ*_detection_ = 762 nm; (C) Cy-Et in methanol, *λ*_ex_ = 629 nm, *λ*_detection_ = 663 nm; (D) C5TNa in methanol, *λ*_ex_ = 723 nm, *λ*_detection_ = 769 nm; (E) C5T-ET (part of Cy-Et) in methanol, *λ*_ex_ = 629 nm, *λ*_detection_ = 656 nm; (F) C5T-ET (part of C5T) in methanol, *λ*_ex_ = 723 nm, *λ*_detection_ = 768 nm.

**Table S5** Fitting Parameters from TA Kinetics.

| **Dyes** | **Signal** | **Wavelength (nm)** | **τ_1_ (ps)** | **A_1_ (%)** | **τ_2_ (ps)** | **A_2_ (%)** |
| --- | --- | --- | --- | --- | --- | --- |
| Cy-Et | ESA | 450 | 427.4 | 100 |  |  |
|  | GSB | 660 | 403.6 | 100 |  |  |
| C5TNa | ESA | 495 | 1.4 | 12 | 439.7 | 87 |
|  | GSB | 755 | 1.2 | 20 | 414.9 | 72 |
| C5T-ET | ESA | 495 | 1.7 | 52 | 23.0 | 38 |
|  | GSB | 660 | 2.9 | 55 | 22.6 | 35 |

A_1_ and A_2_ represent the fraction of excited population of the associated component.


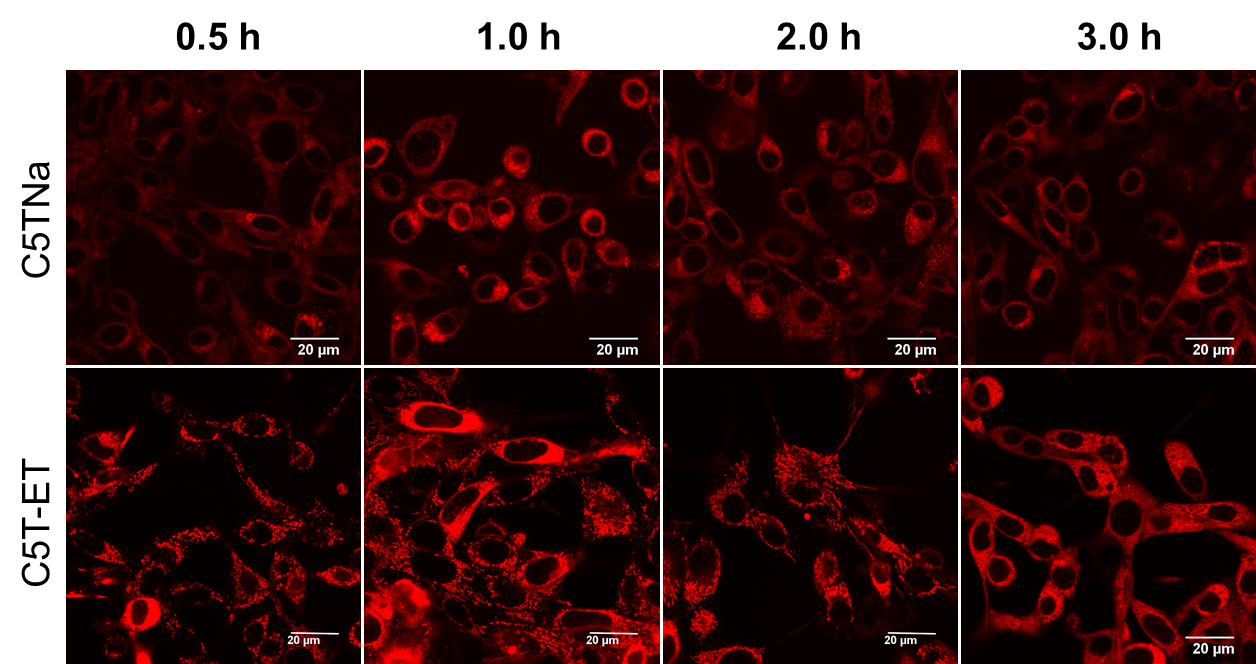


**Figure S13.** Cellular uptake of **C5TNa** and **C5T-ET** in 4T1 cells (2.0 μM, scale bars: 20 μm).

###
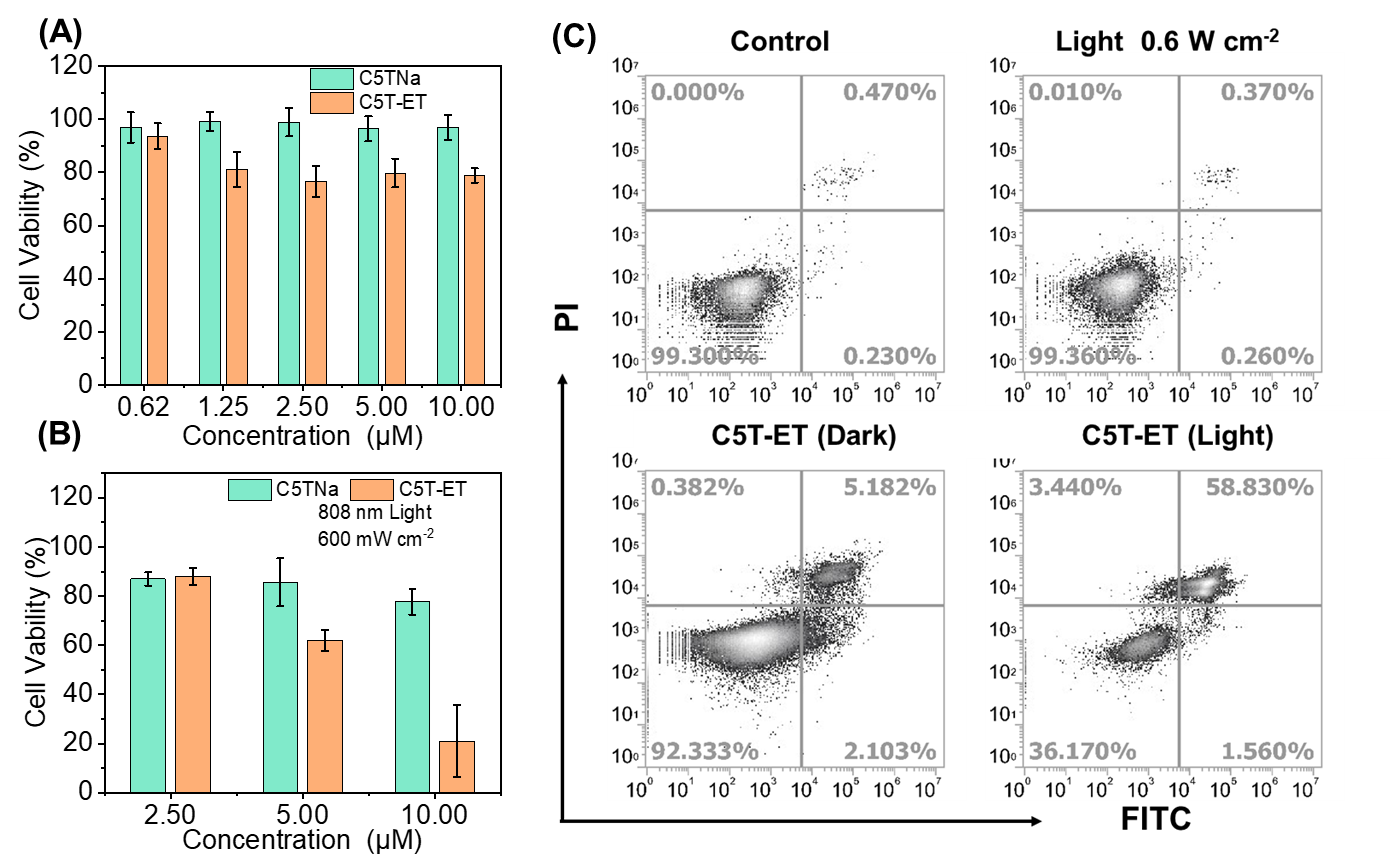


### **Figure S14**. (A, B) Cell viability of 4T1 cells incubated with different concentrations of C5TNa or C5T-ET, with or without laser irradiation. Data are presented as mean±SD (n = 4). (C) Flow cytometry analysis by Annexin V-FITC and PI staining of 4T1 cells following different treatments. Cells were incubated under various conditions and divided into four groups, including (1) Control (without any treatment), (2) 808 nm laser irradiation (5 min, 0.6 W cm^−2^), (3) **C5T-ET** (7.5 μM) without laser irradiation, (4) **C5T-ET** (7.5 μM) + 808 nm laser irradiation (5 min, 0.6 W cm^−2^).


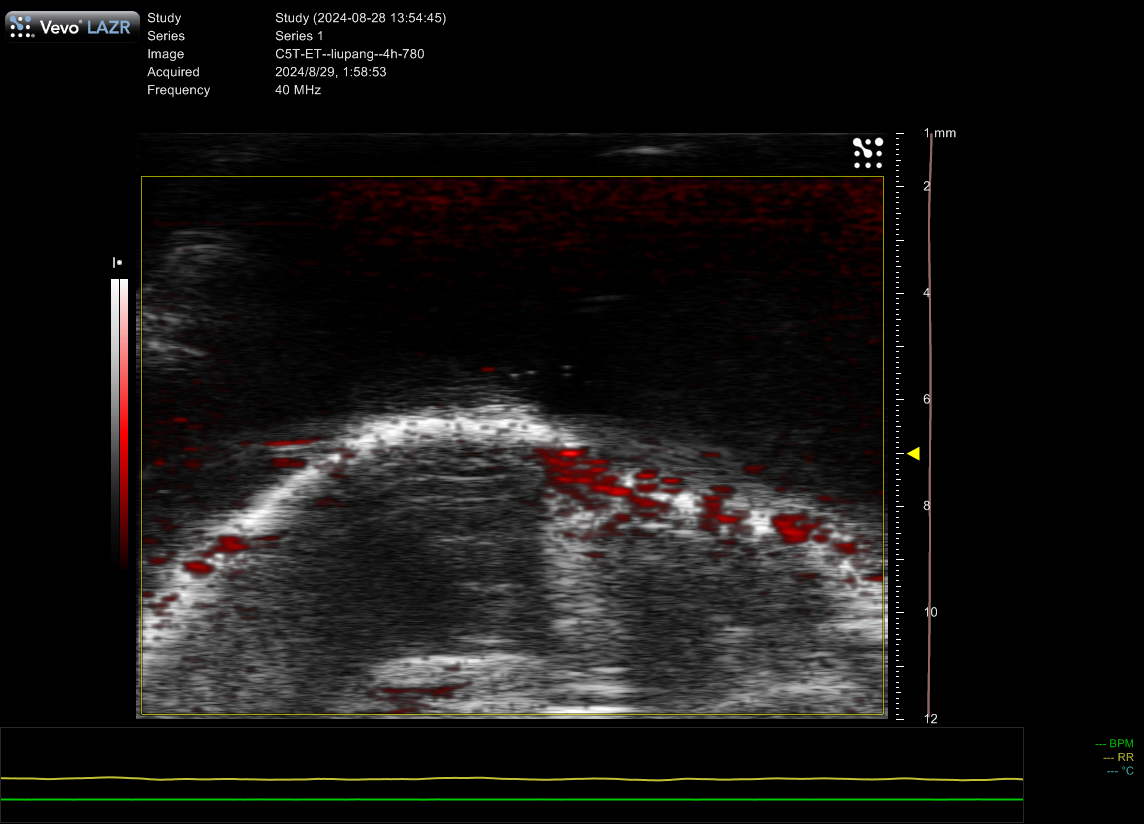


**Figure S15.** PA images of tumor-bearing mouse without treatment under laser excitation.


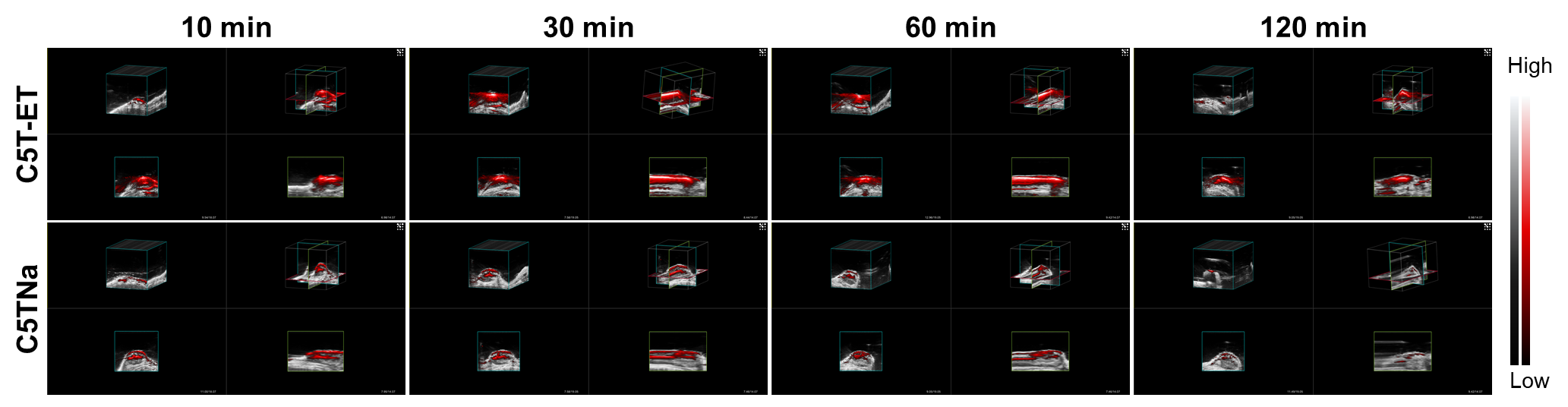


**Figure S16.** 3D-rendered images of the NIR PA signals of C5T-ET and C5TNa.


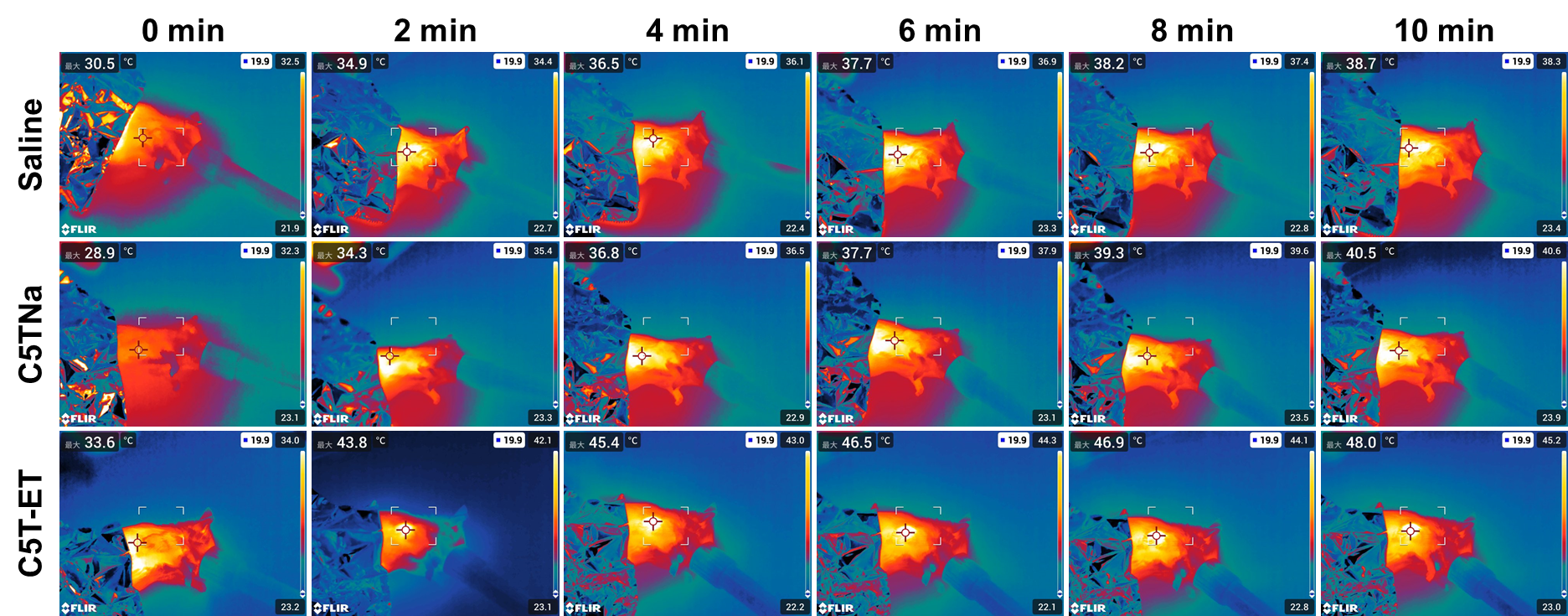


**Figure S17.** Real-time photothermal imaging of 4T1 tumor-bearing BALB/c mice after intratumoral injection of **Saline, C5TNa** and **C5T-ET** recorded at different times. Saline, 100 μL-treated as the control; **C5TNa** (200 μM, 100 μL); **C5T-ET** (200 μM, 100 μL).

**Figure S18.** Temperature changes of 4T1 tumor-bearing mice after various treatments upon 808 nm laser (0.6 W cm^−2^) irradiation. Error bars, mean ±S.D. (n = 3). ***p < 0.001, Statistical significance was determined using ANOVA.


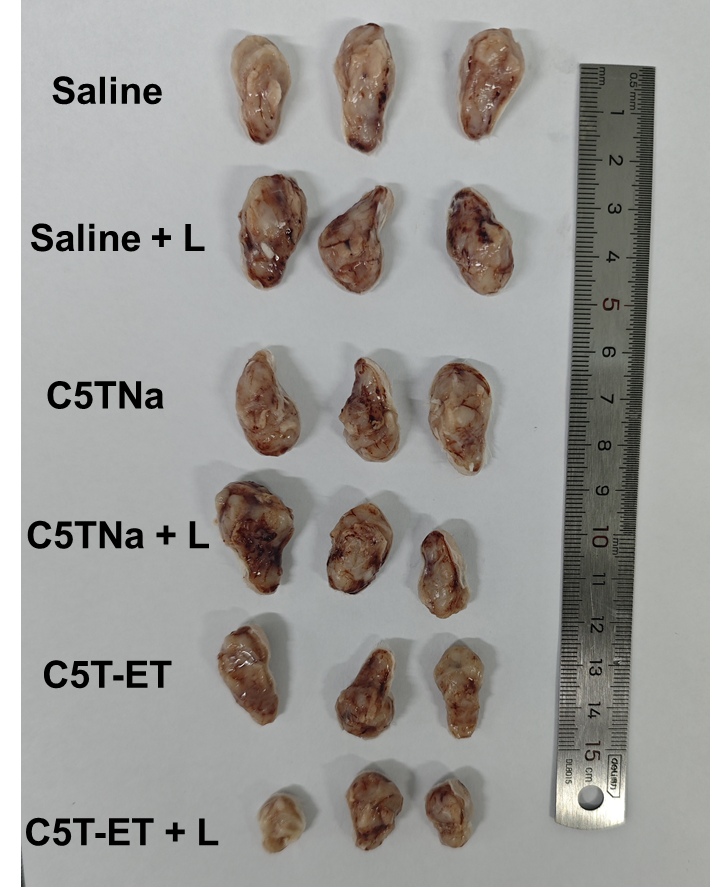


**Figure S19.** Photographs of tumor tissue excised from mice in different groups after treatment.


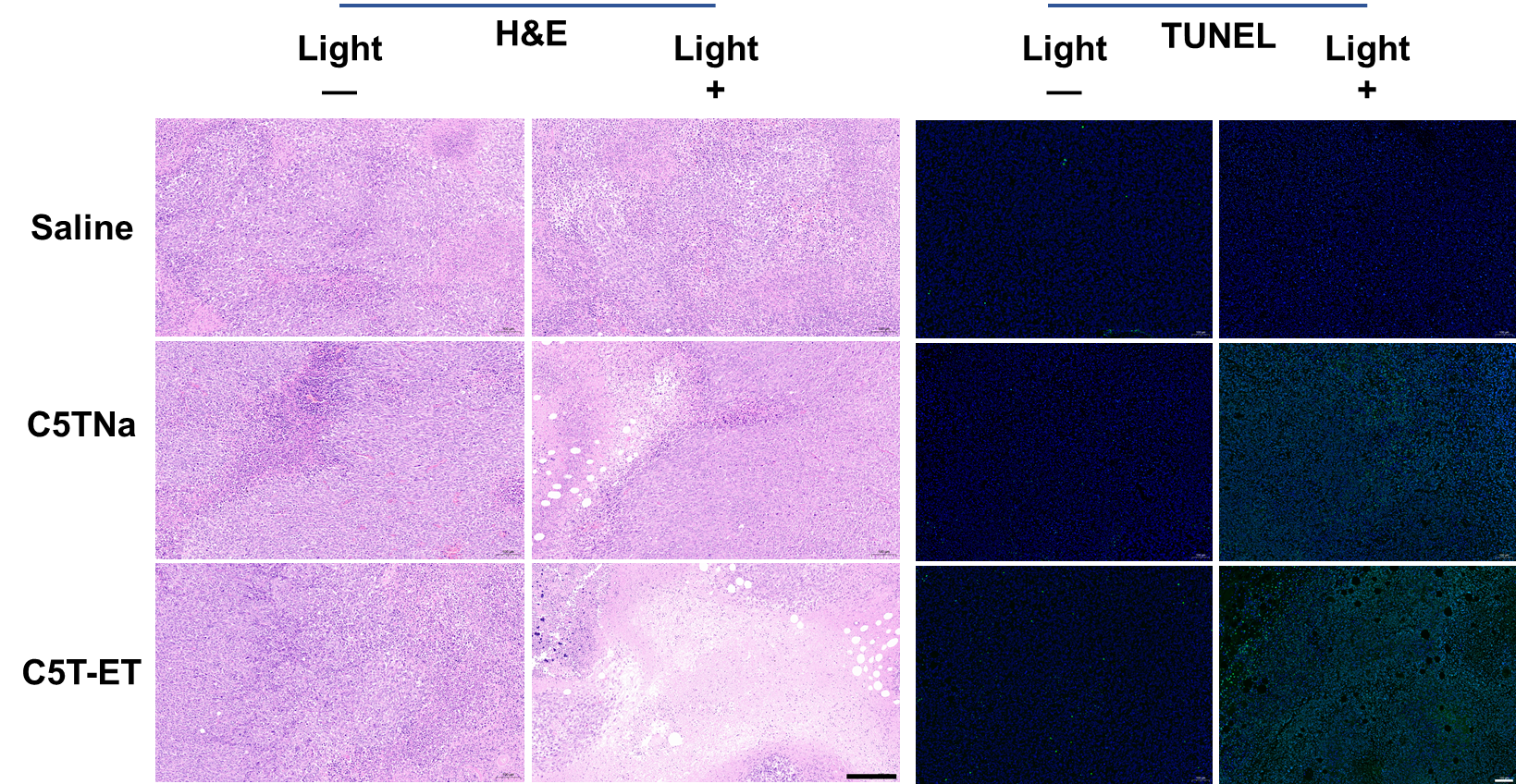


**Figure S20.** H&E and TUNEL staining analysis of tumor tissue from different groups after various treatment (scale bar = 100 μm).


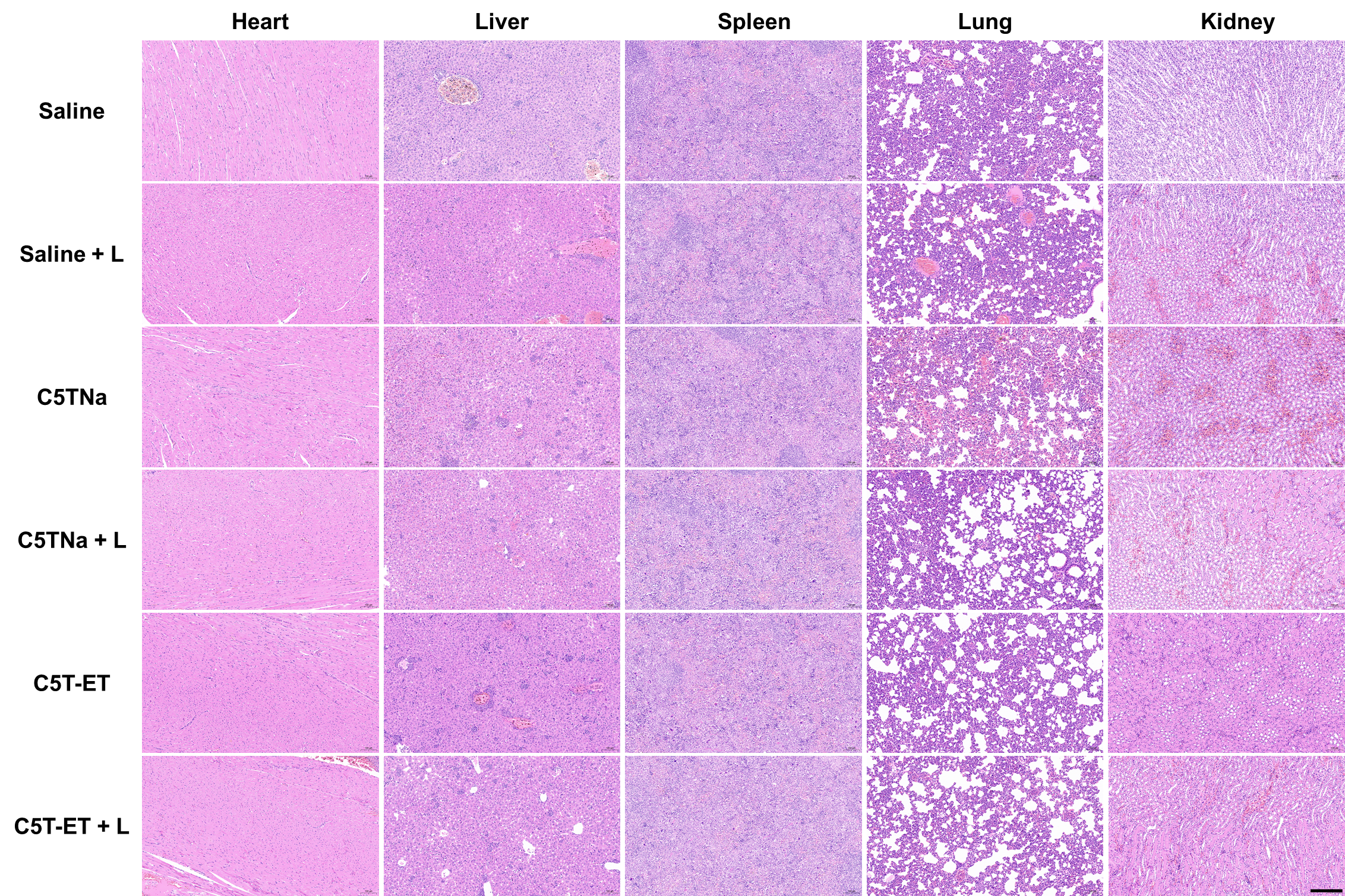


**Figure S21.** H&E stained images of the main organs (heart, liver, spleen, lung, and kidney) of different groups, respectively (scale bar: 200 μm).

**Figure S22.** HRMS spectrum of **C5TNa.**

^^

**Figure S23.** ^1^H NMR spectrum of **C5TNa** in Acetone-d6.

**Figure S24.** ^13^C NMR spectum of **C5TNa** in Acetone-d6.

References:

1. D. Xi, M. Xiao, J. Cao, L. Zhao, N. Xu, S. Long, J. Fan, K. Shao, W. Sun, X. Yan, X. Peng, *Adv. Mater.* **2020**, 32, 1907855.

2. H. Huang, S. Long, M. Li, F. Gao, J. Du, J. Fan, X. Peng, *Dyes Pigment.* **2018**, 149, 633.

3. a)D. Oushiki, H. Kojima, Y. Takahashi, T. Komatsu, T. Terai, K. Hanaoka, M. Nishikawa, Y. Takakura, T. Nagano, *Anal. Chem.* **2012**, 84, 4404; b)C. Sun, S. Lv, Y. Liu, Q. Liao, H. Zhang, H. Fu, J. Yao, *J. Mater. Chem. C* **2017**, 5, 1224.

4. G. Cheng, J. Fan, W. Sun, J. Cao, C. Hu, X. Peng, *Chem. Commun.* **2014**, 50, 1018.

5. Z. An, C. Zheng, Y. Tao, R. Chen, H. Shi, T. Chen, Z. Wang, H. Li, R. Deng, X. Liu, W. Huang, *Nat. Mater.* **2015**, 14, 685.
